# Loop L1 governs the DNA-binding specificity and order for RecA-catalyzed reactions in homologous recombination and DNA repair

**DOI:** 10.1093/nar/gku1364

**Published:** 2015-01-05

**Authors:** Takeshi Shinohara, Shukuko Ikawa, Wakana Iwasaki, Toshiki Hiraki, Takaaki Hikima, Tsutomu Mikawa, Naoto Arai, Nobuo Kamiya, Takehiko Shibata

**Affiliations:** 1 Cellular & Molecular Biology Unit, RIKEN, 2-1 Hirosawa, Wako-shi, Saitama 351-0198, Japan; 2 Advanced Catalysis Research Group, RIKEN Center for Sustainable Resource Science, Wako-shi, Saitama 351-0198, Japan; 3 Department of Supramolecular Biology, Graduate School of Nanobiosciences, Yokohama City University, 1-7-29 Suehiro-cho, Tsurumi-ku, Yokohama, Kanagawa 230-0045, Japan; 4 Advanced Photon Technology Division, Research Infrastructure Group, RIKEN SPring-8 Center, RIKEN Harima Institute, 1-1-1 Kouto, Sayo, Hyogo 679-5148, Japan; 5 Department of Applied Biological Science, Nihon University College of Bioresource Sciences, 1866 Kameino, Fujisawa-shi, Kanagawa 252-8510, Japan

## Abstract

In all organisms, RecA-family recombinases catalyze homologous joint formation in homologous genetic recombination, which is essential for genome stability and diversification. In homologous joint formation, ATP-bound RecA/Rad51-recombinases first bind single-stranded DNA at its primary site and then interact with double-stranded DNA at another site. The underlying reason and the regulatory mechanism for this conserved binding order remain unknown. A comparison of the loop L1 structures in a DNA-free RecA crystal that we originally determined and in the reported DNA-bound active RecA crystals suggested that the aspartate at position 161 in loop L1 in DNA-free RecA prevented double-stranded, but not single-stranded, DNA-binding to the primary site. This was confirmed by the effects of the Ala-replacement of Asp-161 (D161A), analyzed directly by gel-mobility shift assays and indirectly by DNA-dependent ATPase activity and SOS repressor cleavage. When RecA/Rad51-recombinases interact with double-stranded DNA before single-stranded DNA, homologous joint-formation is suppressed, likely by forming a dead-end product. We found that the D161A-replacement reduced this suppression, probably by allowing double-stranded DNA to bind preferentially and reversibly to the primary site. Thus, Asp-161 in the flexible loop L1 of wild-type RecA determines the preference for single-stranded DNA-binding to the primary site and regulates the DNA-binding order in RecA-catalyzed recombinase reactions.

## INTRODUCTION

Homologous genetic (or DNA) recombination plays essential roles in DNA double-strand break repair to maintain genome stability and in genetic diversification, and defects in homologous recombination in mitotic cells cause carcinogenesis and various diseases (see (1–4) for reviews). Homologous joint formation is a crucial step in homologous recombination. In all organisms, this is catalyzed in an adenosine triphosphate (ATP)-dependent manner by the RecA-family recombinases, including bacterial RecA and eukaryotic Rad51 and Dmc1, among which *Escherichia coli* (*E. coli*) RecA has been most thoroughly studied for decades ((5,6); see (7–9) for reviews).

Except for the RecA from *Deinococcus radiodurans*, a bacterium with extreme radiation resistance (10), RecA-family recombinases first bind cooperatively around single-stranded DNA in the presence of ATP (primary DNA binding) to form extended helical filaments (11–14), and consequently unfold some secondary structures of the DNA (see (15)). The primary single-stranded DNA binding activates the ATP hydrolysis activity of RecA (16), as well as the ability to bind to double-stranded DNA in a sequence homology-independent manner (secondary DNA binding). Within this ternary complex of ATP-bound RecA, single-stranded DNA and double-stranded DNA, homologous sequences are searched for without ATP hydrolysis and homologous joints are formed between the two DNAs (17–19). ATP-hydrolysis-dependent strand-exchange then follows (see the Results section). However, if RecA interacts with double-stranded DNA first, then homologous joint formation is prevented. Rad51 also shares the same biochemical characteristics and homologous joint formation mechanism (see (20)). The basic molecular structures are well conserved among the RecA/Rad51 recombinases, which have a structurally well-conserved central domain with the ATP-binding site and two disordered loops, L1 and L2, suggesting common mechanisms of homologous joint formation by these recombinases.

RecA, consisting of 352 amino-acid residues, contains three domains: an N-terminal domain, a central core domain and a C-terminal domain (21). The central domain has the catalytic center for ATP hydrolysis, and ATP binding to RecA and its hydrolysis induce substantial structural alterations (21,22) that seem to play roles in homologous joint formation and following joint processing, including branch migration. The central domain has two flexible loops, L1 (Gly-157 to Met-164) and L2 (Ile-195 or Gln-194 to Thr-209), which could not be modeled in the free RecA crystal structure determined by Steitz's group (21). Loops L1 and L2 constitute the binding sites for the single-stranded or double-stranded DNA cofactor that induces the ATP hydrolysis activity of RecA, as shown by a recent crystallographic analysis of ADP-AlF_4_-Mg^2+^-bound RecA–DNA complexes (‘activated RecA–DNA complex’; 22). In the RecA–DNA complex crystals, loops L1 and L2 are both ordered, and they form a short helix (αL1) followed by a turn and an extended segment, and a β-hairpin (β1_L2_–β2_L2_), respectively. The bases or base pairs of the bound DNA form triplets, which are sandwiched between adjacent L2 β-hairpins (22). The triplets in the RecA-single-stranded DNA crystal and the solution structure of single-stranded DNA bound to RecA have very similar structural features, which clearly differ from B-form DNA and are favorable for complementary sequence recognition between single-stranded and double-stranded DNAs (23).

The roles of loops L1 and L2 beyond DNA binding in homologous joint formation are not well understood. We solved the crystal structure of an inactive DNA-free form of RecA, in which we were able to trace both loops L1 and L2. A comparison of the structures of loop L1 in this RecA and the activated RecA–DNA complexes suggested the regulatory role of loop L1 in homologous joint formation. The biochemical analyses of the mutant recAs highlighted the important role of the aspartate at position 161 (Asp-161) in loop L1, in selecting single-stranded DNA for the primary DNA-binding step by RecA at the start of homologous joint formation.

## MATERIALS AND METHODS

### Media

LB (liquid) contained 1% Bacto-tryptone, 0.5% Bacto-yeast extract and 1% NaCl at pH 7.5; LB plates contained 1.5% agar in LB (liquid) medium. M9 buffer contained 0.1-g NH_2_Cl, 0.3-g KH_2_PO_4_ and 0.6-g Na_2_HPO_4_·7H_2_O per 100 ml of distilled water.

### 90-nucleotide single-stranded DNA

The sequence of the 90-nucleotide single-stranded DNA used in the homologous joint formation by RecA is 5′-AAATCAATCT AAAGTATATA TGAGTAAACT TGGTCTGACA GTTACCAATG CTTAATCAGT GAGGCACCTA TCTCAGCGAT CTGTCTATTT-3′, which has least secondary structures and is the complementary sequence from positions 1932 to 2022 of the pBluescript SK(-) double-stranded DNA (24). This 90-nucleotide single-stranded DNA and that labeled with FAM (6-carboxyfluorescein) at the 5’-terminus were purchased from Eurofins Genomics K.K.

Amounts of DNA are expressed in nucleotides, unless otherwise stated.

### DNA preparation, 5′-labeling of single-stranded DNA, DNA constructs for mutant recA expression, purification of wild-type RecA (RecA-wt) and mutant recAs and crystallization

These are described in the Supplementary data. It is advised to use freshly purified RecA preparation from freshly grown *E. coli* cells, since we experienced some experimental difficulties with the wild-type RecA purified from old 60% glycerol stock of partially purified RecA in a freezer (see Supplementary Figure S9).

### Assay for methyl methanesulfonate sensitivity

*E. coli* MV1184 (*ara*, Δ(*lac-proAB*), *rpsL, thi* (Φ80 *lacZΔ*M15), Δ(*srl-recA*) 306::Tn10 *(tet^r^)*/F′[*traD*36, *proAB*^+^*, lacI*^q^*, lacZ*ΔM15]) cells, with the pKK223-3 plasmid bearing *recA* under the control of the *tac* promoter, or with the empty pKK223-3 plasmid, were cultured at 37°C in 1 ml of LB medium containing 50-μg/ml ampicillin and 10-μg/ml tetracycline for ∼18 h (overnight).

For spot tests, 20-μl portions of the overnight cultures were inoculated into 1 ml of fresh LB medium containing the same amounts of antibiotics, and shaken at 37°C. When the cells reached early stationary phase (OD_600nm_ 2.3–2.6; GE Ultrospec 2100 pro spectrophotometer), the cell culture was diluted with M9 buffer to 1.0 at OD_600nm_, and 5.0-μl portions of the diluted cell culture were spotted on LB agar plates containing 50-μg/ml ampicillin, 10-μg/ml tetracycline and the indicated amounts of methyl methanesulfonate (MMS). The spotted plates were incubated at 37°C for the indicated times.

For a quantitative assay, 20-μl aliquots of the overnight cultures were inoculated into 2.0 ml of fresh LB medium containing the same amounts of antibiotics, and shaken at 37°C for 6 h. Each cell culture was diluted appropriately for colony counts and spread on LB agar plates containing 50-μg/ml ampicillin, 10-μg/ml tetracycline and MMS (0.58, 1.7, 3.5, 7.0, 9.3 or 11.7 mM). The plates were incubated at 37°C for 1 or 2 days, as indicated. The numbers of colonies formed on the plates were counted.

### Standard reaction buffer

The standard reaction buffer consisted of 31-mM Tris-HCl (pH 7.5), 13-mM MgCl_2_, 1.3-mM ATP, 1.8-mM dithiothreitol and 88-μg/ml bovine serum albumin (Boehringer-Mannheim, for molecular biology), as described previously (25,26), unless otherwise stated.

### Assay for DNA binding by electrophoresis mobility shift assay

For single-stranded DNA binding, the 90-nucleotide single-stranded DNA (5.0-μM unlabeled and 0.1-μM ^33^P-labeled at the 5′ terminus) was incubated with the indicated amounts of RecA in a 10-μl standard reaction buffer at 37°C for 5 min and fixed by 0.01% fresh glutaraldehyde at 37°C for 3 min. The samples were mixed with 1/10 volume of loading buffer B (50% glycerol, 0.05% each of bromophenol blue, xylene cyanol and orange G, no ethylenediaminetetraacetic acid (EDTA)), loaded onto a 1.2% agarose gel, and electrophoresed in 0.5x TBE buffer (25-mM Tris, 24-mM boric acid, 1-mM EDTA, pH 8.0) at 50 V/12.5 cm for 75 min at room temperature. After electrophoresis, the gel was dried and the ^33^P-signals were analyzed with a BAS-2000 or BAS-2500 image analyzer (Fuji Photo Film Co., Ltd.). In some experiments, the 90-nucleotide single-stranded DNA was labeled at 5′ terminus with FAM, and analyzed by a LAS-1000 Luminescent Image Analyzer (Fuji Photo Film Co., Ltd.).

For double-stranded DNA-binding, pBluescript SK(-) double-stranded DNA (18 μM, closed circular form) was incubated with the indicated amounts of RecA, in a 10-μl standard reaction buffer at 37°C for 15 min. The samples were then mixed with 1 μl of the loading buffer B and loaded onto a 1.0% agarose gel, and electrophoresed at 40 V/12.5 cm for 120 min at room temperature. The gel was stained with 1-μg/ml ethidium bromide and was photographed with UV illumination at 312 nm. Smart Ladder (from top to bottom, 10, 8, 6, 5, 4, 3, 2.5, 2, 1.5, 1.0, 0.80 and 0.60 Kbps; Nippon Gene) markers were used as DNA size markers.

### Assay for adenosine triphosphatase activities of RecA

Adenosine triphosphatase (ATPase) activities were assayed as described previously (15). In the standard reaction buffer (20 μl), [^14^C or α-^32^P]ATP (26 nmol at 1.3 mM) was incubated with the indicated amounts of RecA, MgCl_2_ and indicated DNA, at 37°C for 30 min. The sample was subjected to thin layer chromatography as described (15), but the unlabeled adenosine triphosphate (ATP), adenosine diphosphate (ADP) and adenosine monophosphate (AMP) markers were omitted. The amounts of radioactively labeled ATP, ADP and AMP were determined with a BAS-2000 or BAS-2500 image analyzer, and the amounts of ATP hydrolyzed by the reaction were calculated.

### Assay for LexA cleavage

M13 single-stranded DNA or pBluescript SK(-) double-stranded DNA (18 μM) and RecA (1.0 μM) were first incubated at 37°C for 20–30 min in the standard reaction buffer, except with 5.0-mM MgCl_2_, containing an ATP-regenerating system. The ATP-regenerating system used in this study consisted of 4.8-mM phosphocreatine and 7.8 units/ml of creatine phosphokinase (Sigma Chemical Co.). LexA cleavage was initiated by the addition of LexA repressor protein (4.7 μM) to the reaction mixture (final, 21 μl), followed by an incubation at 37°C. At the indicated times, 5-μl aliquots were withdrawn. The samples were mixed with 5-μl 2×SDS sample buffer (125 mM Tris-HCl (pH 6.8), 10% 2-mercaptoethanol, 4% sodium dodecyl sulphate (SDS), 10% sucrose, 0.01% bromophonol blue (BPB)) at 0°C, and then heated at 95°C for 5 min to terminate the reaction. The products and unreacted substrates were then separated by electrophoresis through a 15% polyacrylamide gel containing 0.4% SDS. The gel was stained by 0.25% Coomassie Brilliant Blue and photographed. Precision Plus Protein™ All Blue Standards (250, 150, 100, 75, 50, 37, 25, 20, 15 and 10 kDa), purchased from Bio-Rad Laboratories, Inc., were used as protein size markers

### Assay for homologous joint formation (D-loop assay) by RecA

Under the standard conditions for homologous joint formation, the 90-nucleotide single-stranded [^33^P]DNA (0.05 μM) and RecA (2.0 μM) were first incubated at 37°C for 10 min, in the standard reaction buffer containing the ATP-regenerating system. The ATP-regenerating system used in this study consisted of 4-mM phosphocreatine and 5 units/ml of creatine phosphokinase (Sigma Chemical Co.). Homologous joint formation was initiated by the addition of homologous form I (negatively supercoiled closed circular double-stranded) DNA (pBluescript SK(-); 18 μM) to the reaction mixture (final, 21 or 41 μl), followed by an incubation at 37°C. At the indicated times, 4.5-μl aliquots were withdrawn. The samples were mixed with 1.7-μl 66.7-mM EDTA and 5% SDS solution on ice to terminate the reaction and were treated with 2.4-mg/ml (final concentration) Proteinase K at 0°C or 37°C for 15–45 min to remove the proteins. The samples were mixed with 1/10 volume of 10x loading buffer H (0.9% SDS, 50% glycerol, 0.05% each of bromophenol blue, xylene cyanol and orange G), and the products and unreacted substrates were then separated by 1.0% agarose gel electrophoresis in 0.5x TBE buffer (25-mM Tris, 24-mM boric acid, 1-mM EDTA, pH 8.0). After the gel was dried, the ^33^P-signals were analyzed quantitatively with a BAS-2000 or BAS-2500 image analyzer (Fuji Photo Film Co., Ltd.). The Proteinase K treatment at 0°C yielded somewhat increased amounts of the RecA-catalyzed homologous joint-formation products, as compared to the treatment at 37°C, probably because of the heat instability of some of the homologous joints, as observed previously (27).

## RESULTS

### RecA filament and loops L1 and L2 in the crystal

We crystallized free *E. coli* RecA (indicated as ‘RecA’) in the *P*6_1_ space group and determined the structure at 2.8-Å resolution (Supplementary Table S1; PDB ID: 4TWZ). The RecA helical filaments in the crystals had six protomers per turn, with a 72.1-Å helical pitch (Supplementary Figure S1A, left panel), suggesting that this crystallographic filament reflects an inactive state (see Supplementary Figure S1). The core and the C-terminal domains in each protomer of the inactive DNA-free RecA and those of the activated RecA–DNA complexes (22) superimposed well (root-mean-square deviation of 1.03 Å for Cαs of residues 45–156, 165–194 and 210–328), but the orientations of the N-terminal domains and the conformations of loops L1 and L2 significantly differed. The details of the crystallization and some features of the crystal structure are described in the Supplementary data.

**Figure 1.**
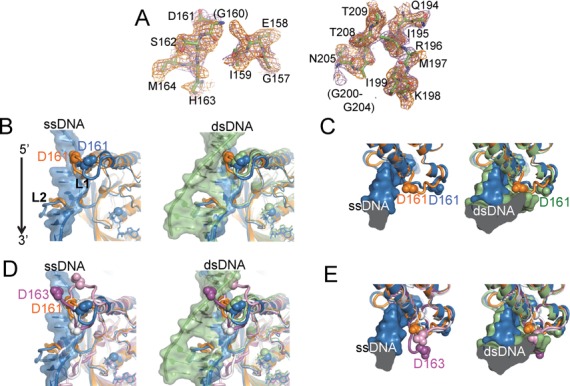
Comparison of loops L1 and L2 in RecA crystals. (**A**) Electron densities for loops L1 and L2 in the inactive RecA crystal that we determined. Sigma-A-weighted simulated annealing *F*_o_-*F*_c_ omit map of loops L1 and L2 (2.2σ, orange) and the 2*F*_o_-*F*_c_ map (0.7σ, purple). (**B**) Left panel: comparison of loops L1 and L2 in the structures of the inactive RecA (orange) and the activated RecA-single-stranded DNA complex (22) (blue). Right panel: the structure of the activated RecA-double-stranded DNA complex is also superimposed (green). D161 in loop L1 is depicted by a cpk model. (**C**) Top view of panels in (B). (**D**) The structures of the free msRecA (white) and the msRecA-ADP (pink) (36) and msRecA-dATP (magenta) (38) complexes are further superimposed on panel (B). (**E**) Top view of panels in (D).

Unlike a number of previously reported crystals of RecA in the absence of DNA, our inactive RecA crystal provided an electron density map for most of both loops L1 and L2 (Figure 1A). A comparison with the structures of the activated RecA–DNA complexes (22) suggested that some prominent conformational changes occur in these loops upon DNA binding. While loop L1 in the inactive RecA is unfolded, in the activated RecA–DNA complexes it forms a short helix (αL1) followed by a turn and an extended segment. Asp-161 lies at the tip of loop L1 (Figure 1A). In the structure of free *E. coli* RecA, Asp-161 is located at a position where it would not obstruct single-stranded DNA binding (left panels of Figure 1B and C). On the other hand, the spatial location of Asp-161 in the free RecA causes steric clash with and electrostatic repulsion to a complementary DNA strand that was located in the structure of the RecA-double-stranded DNA complex (22) (right panels of Figure 1B and C). Thus, the flexible loop L1 appears to cause a steric barrier to a complementary DNA strand. Therefore, we studied the effects of the amino-acid replacements of Asp-161 on the activities of RecA.

### The D161A substitution enhanced double-stranded DNA binding, with slight effects on single-stranded DNA binding

**Figure 2.**
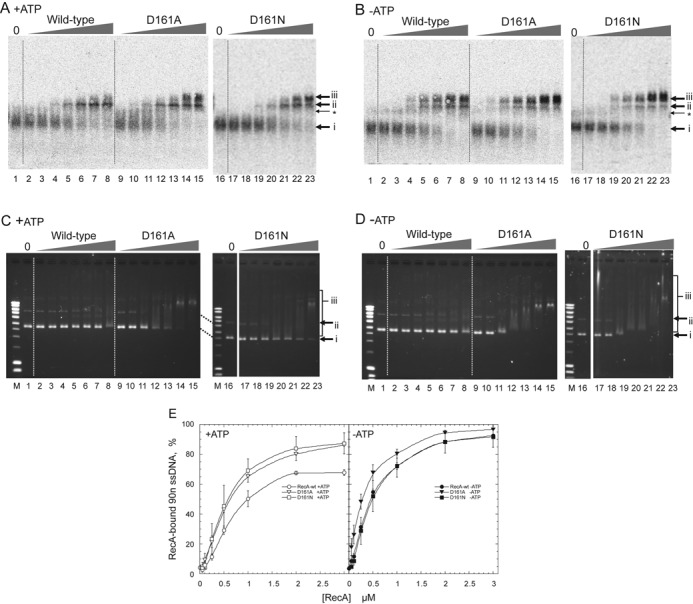
Amino-acid replacement of Asp-161 by Ala (D161A) or Asn (D161N) in loop L1 enhances double-stranded DNA-binding, but not single-stranded DNA-binding. (**A, B**) Single-stranded DNA-binding. 90-nucleotide single-stranded [^33^P]DNA (0.5 μM) was incubated with the indicated amounts of mutant or wild-type RecA for 5 min in the presence (A) or absence (B) of ATP in the standard reaction buffer for homologous joint formation. The samples were then fixed and subjected to agarose gel electrophoresis. The radioactivity in the gel was analyzed. Amounts of RecA in (A) and (B): lanes 1 and 16, no RecA; lanes 2, 9 and 17, 0.05 μM; lanes 3, 10 and 18, 0.1 μM; lanes 4, 11 and 19, 0.25 μM; lanes 5, 12 and 20, 0.5 μM; lanes 6, 13 and 21, 1.0 μM; lanes 7, 14 and 22, 2.0 μM; lanes 8, 15 and 23, 3.0 μM. Arrows i, unbound single-stranded DNA; ii and iii, RecA-bound single-stranded DNA. *Two single-stranded DNA-molecules that would interact with each other by local base-pairing. (**C, D**) Double-stranded DNA-binding. Unlabeled pBluescript SK(-) supercoiled (form I) double-stranded DNA (18 μM) was incubated for 15 min with the indicated amounts of mutant or wild-type RecA in the presence (C) or absence (D) of ATP in the standard reaction buffer for homologous joint formation. The samples were then subjected to agarose gel electrophoresis without fixation. The gel was stained with ethidium bromide and the DNA signals were detected by UV irradiation. Contrast was enhanced. Amounts of RecA: lanes 1 and 16, no RecA; lanes 2, 9 and 17, 0.5 μM; lanes 3, 10 and 18, 1.0 μM; lanes 4, 11 and 19, 2.0 μM; lanes 5, 12 and 20, 3.0 μM; lanes 6, 13 and 21, 4.0 μM; lanes 7, 14 and 22, 5.0 μM; lanes 8, 15 and 23, 10 μM. Lane M: DNA size markers. Arrows i, free form I; ii, nicked circular double-stranded DNA present in the form I preparation; iii, RecA-bound double-stranded DNA. (**E**) Quantitative representation of single-stranded DNA-binding analysis. The unbound 90-nucleotide single-stranded DNA among the total DNA signals in each lane was quantified, and the value of 100 minus% unbound single-stranded DNA was plotted as% RecA-bound single-stranded DNA (90n ssDNA) against the amounts of RecA. Each point indicates the average obtained from three independent experiments. ▽ (open inverse triangles), ▼ (closed inverse triangles), recA-D161A; ◻ (open square), ◼ (closed square), recA-D161N; ○ (open circles), ● (closed circles), RecA-wt. Open symbols, with ATP; closed symbols, without ATP.

We constructed an expression vector and purified the mutant recA with the Ala- and Asn-substitutions of Asp-161 (recA-D161A and recA-D161N, respectively). Before we tested whether these replacements of Asp-161 (D161A) stimulated double-stranded DNA binding by an electrophoresis mobility shift assay, we analyzed the effects of the D161A and D161N substitutions on single-stranded DNA binding, using the 90-nucleotide single-stranded [^33^P] DNA with the least secondary structure (see the sequence shown in the Materials and Methods section). This 90-nucleotide single-stranded DNA was prepared for a homologous joint-formation assay, with minimal effects of the secondary structure. After the incubation of the 90-nucleotide single-stranded [^33^P] DNA under the standard buffer conditions for homologous joint formation, the samples were fixed by glutaraldehyde and subjected to agarose gel electrophoresis. The intensity of the free DNA signal (arrow i) was decreased, with a concomitant increase in the signal of RecA-bound DNA, along with the increase in the amount of either recA-D161A or recA-D161N, or of the wild-type RecA (RecA-wt) in the presence or absence of ATP (arrows ii and iii; Figure 2A and B). The D161A and D161N substitutions only slightly increased, if at all, the binding of RecA to the single-stranded DNA, in the presence of ATP (Figure 2E; 1.2-fold, compared by the protein amounts required to achieve half-maximum binding; see Supplementary Figure S2 for details of calculations) and in the absence of ATP (1.6-fold as for D161A, no stimulation as for D161N; see Supplementary Figure S2). Notably, in the presence of ATP, the intensity of signal iii derived from RecA-wt was significantly smaller than that derived from recA-D161A or recA-D161N (Figure 2A and B). Both signals of ii and iii are derived from DNA–RecA complexes with homogeneous sizes but with different conformations. This difference would be induced by the binding of one strand or two strands in the primary DNA-binding site, since the primary site can accommodate two strands and it is predicted that the binding of the second DNA strand at the primary site is prevented by Asp-161 in RecA-wt.

Since we intended to understand how RecA selected single-stranded DNA at the start of homologous joint formation, we analyzed DNA binding by use of 90-nucleotide single-stranded DNA as described above and double-stranded DNA (form I DNA), both of which were used for homologous joint formation. Form I DNA was incubated under the same conditions and analyzed by agarose gel electrophoresis without fixation. As expected from the assumption that Asp-161 inhibits the binding of the second DNA strand, the D161A substitution clearly enhanced double-stranded DNA (form I DNA) binding, in either the presence or absence of ATP (3–5-fold, determined by comparing the protein amounts giving similar mobility shift extents; Figure 2C and D). To test whether the loss of the negative charge or the reduced size of side chain of Asp-161 is responsible to the observed enhancement, we analyzed the effects of the asparagine substitution of Asp-161. The D161N also enhanced form I DNA binding either in the presence or absence of ATP, but in the presence of ATP, the extent of the enhancement was smaller than the D161A substitution (Figure 2C and D). Thus, Asp-161 is responsible for the selectivity toward single-stranded DNA in DNA binding by wild-type RecA, by its negative charge and in the presence of ATP, partly by its mass.

We repeated the DNA binding assays with the 90-nucleotide single-stranded DNA and the form I DNA in the presence of 1.0-mM MgCl_2_, the optimal conditions for form I-dependent ATPase activity of RecA, instead of the standard conditions (13-mM MgCl_2_) for homologous joint formation. The stimulation of double-stranded DNA binding and a slight effect on single-stranded DNA binding of RecA by D161A and D161N substitutions were not significantly altered by changing MgCl_2_ concentrations (Supplementary Figure S3).

Although the D161A substitution extensively stimulated double-stranded DNA binding, recA-D161A still showed higher affinity toward single-stranded DNA than double-stranded DNA, especially in the presence of ATP, as determined by comparing the minimum amounts of the protein required to detect the mobility shift (ca. 0.1 μM versus 2 μM) and those to generate the entire shift (2 μM versus 10 μM; Figure 2A and C).

### The D161A substitution allows various forms of double-stranded DNA to be accepted as a cofactor for ATP hydrolysis

In addition to recA-D161A and recA-D161N, we constructed expression vectors and purified the mutant recA with Glu substitutions for Asp-161 (recA-D161E). We compared the ATP hydrolysis by the mutant recAs and RecA-wt, in the presence of single-stranded DNA or double-stranded DNA, to test their primary DNA-binding abilities which induces the conformational change for ATP hydrolysis.

**Figure 3.**
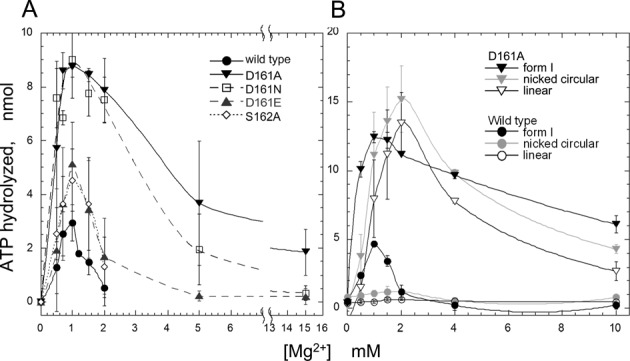
Effects of amino-acid replacements for Asp-161 in loop L1 on the double-stranded DNA-dependent ATPase activity of RecA at various MgCl_2_ concentrations. [^14^C or α-^32^P]ATP (26 nmol at 1.3 mM) was incubated with 2.0-μM mutant recA or RecA-wt in the presence of the indicated amounts of MgCl_2_ and 10-μM pGSat4 (in (**A**)) or 23-μM pBluescript SK(-) (in (**B**)) supercoiled double-stranded DNA (form I DNA). The reaction products were fractionated by thin layer chromatography, and the amounts of ATP hydrolyzed were determined by analyzing the ^14^C- or ^32^P-signals. (A) Effects of the various amino-acid replacements of Asp-161, and the Ala-replacement of Ser-162. The double-stranded DNA was the negatively supercoiled closed circular form (form I), and ^14^C-labeled ATP was used. Amino-acid replacements of mutant recAs used in these experiments are indicated within each panel. Each point indicates the average obtained from two to three independent experiments. (B) Effects of variations in the form of the double-stranded DNA. The double-stranded DNA used was form I (black closed symbols), the nicked circular form (grey open symbols) or the linear form (black open symbols). [α-^32^P]ATP was used in these experiments. ▼, ▽ (inverted triangles), recA-D161A; ●, ○ (circular symbols), RecA-wt. In **(**B) and the following figures, each point indicates the average obtained from more than three independent experiments. Some symbols are larger than the error bars, and thus cover them.

While all of the mutant recAs retained the wild-type level of single-stranded DNA-dependent ATPase activity (Supplementary Figure S4), as expected, the D161A substitution extensively enhanced the double-stranded DNA-dependent ATPase activity, especially in the presence of MgCl_2_ at the concentration required for homologous joint formation (more than 5 mM; (25)), and was immune to the stringent MgCl_2_ concentration requirement (Figure 3A). Under the conditions for ATP-dependent homologous joint formation (standard: neutral pH 7.5, 5–20-mM Mg^2+^), double-stranded DNA is a poor cofactor for ATP hydrolysis by RecA-wt (25,28). RecA-wt exhibited ATP hydrolysis in the presence of negatively supercoiled circular double-stranded DNA (form I DNA), but only in a very narrow range of low Mg^2+^ concentrations around 1 mM ((5,25); Figure 3A).

The D161N replacement showed a similar level of stimulation as the D161A replacement around 1-mM MgCl_2_ but stimulation became smaller at higher concentrations of MgCl_2_ (Figure 3A), as observed by double-stranded DNA-binding assay (Figure 2C and D). The D161E replacement and the S162A substitution had no effect on the MgCl_2_ concentration requirement (Figure 3A). Thus, the stimulation by the D161A substitution is likely to be primarily derived from the absence of a negative charge at this position, and partly by decreased mass of the side chain.

**Figure 4.**
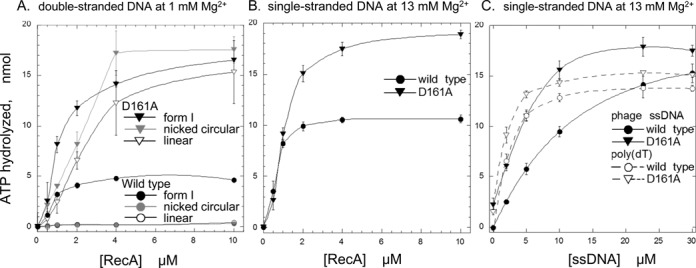
Amino-acid replacement of Asp-161 by Ala (D161A) in loop L1 removed the choice of DNA forms as a cofactor of the ATPase activity of RecA. (**A**) Various forms of double-stranded DNA. [α-^32^P]ATP (26 nmol at 1.3 mM) was incubated with the indicated amounts of recA-D161A or RecA-wt in the presence of a lower concentration (1.0 mM) of MgCl_2_ and 23 μM of various forms of pBluescript SK(-) double-stranded DNA. The amounts of ATP hydrolyzed were measured as described in Figure 3. The forms of double-stranded DNA used were form I (black closed symbols), the nicked circular form (grey open symbols) or the linear form (black open symbols). (**B**) Single-stranded DNA. [α-^32^P]ATP (26 nmol at 1.3 mM) was incubated with the indicated amounts of recA-D161A or RecA-wt in the presence of the standard concentration of MgCl_2_ and 10-μM M13 phage single-stranded DNA. (**C**) Various single-stranded DNAs. [α-^32^P]ATP was incubated with 2.0-μM recA-D161A or RecA-wt in the presence of the standard concentration of MgCl_2_ and the indicated amounts of M13 phage single-stranded DNA (closed symbols) or poly(dT) (open symbols), as in (B). ▼, ▽ (inverted triangles), recA-D161A; ●, ○ (circular symbols), RecA-wt.

The difference in the double-stranded DNA-dependent ATPase activities between the RecA-wt and the recA-D161A was more prominent when the double-stranded DNA lacked supercoils (nicked circular and linear double-stranded DNA). Form I DNA has some characteristics of single-stranded DNA, because its topological stress is favorable for the local melting of a double helix, as shown by the sensitivity to the single-stranded DNA-specific S1 nuclease (29). In the presence of nicked circular or linear double-stranded DNA, RecA-wt only exhibited very limited ATP hydrolysis, but the ATP hydrolysis by recA-D161A was equivalent to that in the presence of form I DNA at various concentrations of MgCl_2_ (Figures 3B and 4A) and reached about half of that in the presence of single-stranded DNA even at 10–13-mM MgCl_2_ (compare Figures 3B and 4C).

These results suggested that recA-D161A can bind single-stranded DNA with secondary structure, which prevents the binding of RecA-wt. Supporting this suggestion, in the presence of a sufficient amount of DNA relative to RecA, the mutant and wild-type RecAs showed similar levels of ATP hydrolysis (Figure 4B and C). At lower concentrations of M13 phage single-stranded DNA relative to the protein, recA-D161A showed higher amounts of ATP hydrolysis than RecA-wt (Figure 4B and C). As expected, when poly(dT), which lacks intramolecular base pairing, was used to replace the single-stranded DNA, no significant difference in the amounts of ATP hydrolysis between the mutant recA and the RecA-wt was detected, even in the presence of limited amounts of poly(dT) (Figure 4C).

### The D161A substitution enables RecA to use double-stranded DNA as a cofactor for LexA repressor cleavage

**Figure 5.**
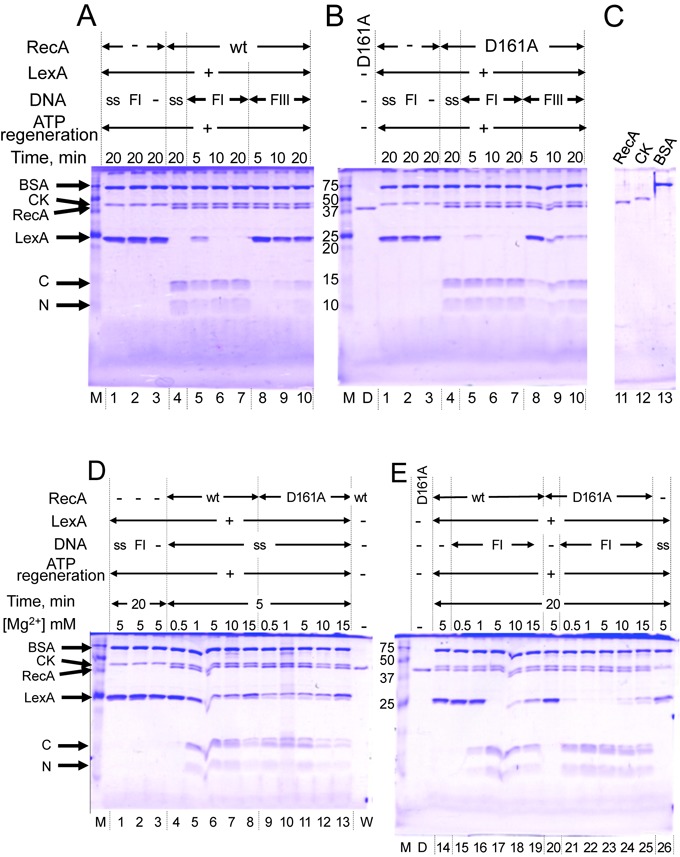
D161A replacement in loop L1 allows RecA to use double-stranded DNA as a cofactor for LexA repressor cleavage. (**A, B, C**) DNA cofactors for LexA cleavage. LexA (4.7 μM) was incubated for the indicated times with 1.0-μM RecA-wt (A) or recA-D161A (B) in the presence of the indicated DNA (18 μM) and 5.0-mM MgCl_2_. (**D, E**) Effects of MgCl_2_ concentrations on LexA cleavage. LexA was incubated for 5 or 20 min (as indicated) with 1.0-μM RecA-wt or recA-D161A in the presence of 18-μM single-stranded DNA (D) or double-stranded DNA (E), in reaction mixtures containing the indicated concentrations of MgCl_2_. DNA added to the reaction mixture: ss, single-stranded DNA; FI, negatively supercoiled closed circular DNA (form I DNA); FIII, linear double-stranded DNA (form III DNA). Proteins in signals were indicated on the left side of the panels: BSA, bovine serum albumin; CK, creatine phosphokinase from the ATP-regenerating system; RecA, RecA-wt or recA-D161A; LexA, uncleaved LexA; C and N, C-terminal and N-terminal fragments, respectively, of cleaved LexA. Lane M: protein markers, with sizes in kDa indicated on the left sides of the gels in (B) and (E); lane D, recA-D161A; lane W, RecA-wt.

RecA prefers single-stranded DNA, rather than double-stranded DNA, as a cofactor for LexA cleavage (30). Negatively supercoiled double-stranded DNA (form I) acted as a cofactor for LexA cleavage by RecA-wt equally well as single-stranded DNA, with the best cleavage in the presence of 5.0-mM MgCl_2_ (Figure 5A and D), but linear double-stranded DNA (form III) did not function well (Figure 5A). While the mutant and wild-type RecAs showed similar LexA-cleavage activities in the presence of single-stranded DNA and various MgCl_2_ concentrations (Figure 5D), recA-D161A cleaved LexA even in the presence of linear double-stranded DNA (Figure 5A and B), and with a much wider range of MgCl_2_ concentrations in the presence of double-stranded DNA (Figure 5E).

Taken together, the results described above indicate that the D161A replacement largely decreased the selectivity toward single-stranded DNA over double-stranded DNA and eliminated the specificity toward negative supercoils in double-stranded DNA for the primary DNA binding by RecA.

### The D161A substitution reduced the suppressed homologous joint formation in the double-stranded DNA preincubation reaction of RecA

To study the direct effects of the mutations on homologous joint formation and subsequent branch migration, in the absence of the effects of the secondary structure of single-stranded DNA, we used the 90-nucleotide single-stranded DNA with the least secondary structure (see the sequence shown in the Materials and Methods section).

We then analyzed the ability of the mutant recAs to form homologous joints and catalyze ATP hydrolysis-dependent branch migration. The consequences of branch migration *in vitro* by RecA-family recombinases depend on the DNA substrates: when the substrates are closed circular double-stranded DNA with negative supercoils and single-stranded DNA-fragments or tails, the RecA- or Dmc1-promoted branch migration results in the dissociation of the homologous joint (31–34). While a stoichiometric amount of RecA to single-stranded DNA is required for the homologous joint formation (27), the dissociation of homologous joints requires an amount of RecA stoichiometric to double-stranded DNA (32). In the cells of all organisms, closed circular double-stranded DNA (or its equivalent form) with a negative writhe is the natural form of circular extrachromosomal double-stranded DNA, called ‘form I’, and of the chromatin DNA loops anchored to chromosomal scaffolds. *In vivo*, the branch migration is associated with repair DNA synthesis, and thus the joint is maintained.

**Figure 6.**
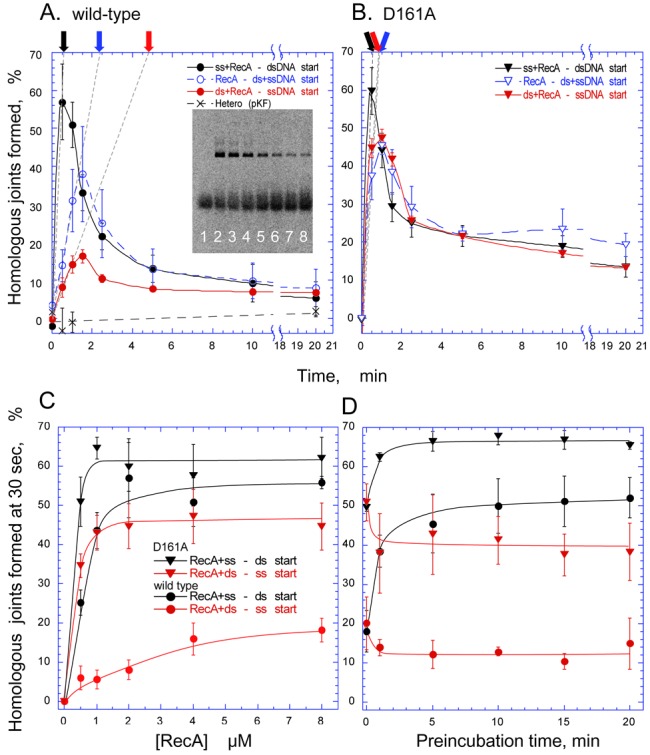
D161A replacement reduced the effects of the order of addition among protein, single-stranded DNA and double-stranded DNA on homologous joint formation. In the standard order of addition, the 90-nucleotide single-stranded [^33^P]DNA (final 0.05 μM) and 2.0-μM RecA-wt or recA-D161A were incubated for 5 min or more (preincubation), and then the reaction was initiated by the addition of homologous (pBluescript SK(-)) form I DNA (18 μM). After an incubation for the indicated times, aliquots were withdrawn and the reaction was terminated. The proteins were removed from the samples, and the reaction products were separated by agarose gel electrophoresis. The ^33^P-signals of products and unreacted substrates were analyzed quantitatively. (**A, B**) The effects of the order of the addition of DNA and RecA. The amounts of homologous joints formed were plotted against the time of incubation, after the initiation of the joint formation by RecA-wt (A) or recA-D161A (B). Inset in (A), ^33^P-image of gel obtained by the image analyzer, for an analysis of the reaction products under the standard conditions with RecA-wt. The reaction times of the samples in the lanes from left to right are 0, 0.5, 1.0, 1.5, 2.5, 5.0, 10 and 20 min. Dashed straight lines with black, blue and red arrows represent the initial velocity of the homologous joint formation in the standard order of addition, the simultaneous addition and the preincubation with double-stranded DNA, respectively. (**C**) The reaction with various amounts of RecA. The formation and dissociation of the homologous joint was measured in the presence of the indicated amounts of proteins (Supplementary Figure S6). The amounts of homologous joints formed within the first 30 s of the incubation were plotted as representatives of the initial velocity of the formation. (**D**) Effects of the preincubation time on homologous joint formation by RecA. Single-stranded DNA or double-stranded DNA was incubated with RecA-wt or recA-D161A for the indicated times. The homologous joint-forming reaction was then started by the addition of the partner DNA. The amounts of homologous joints formed at 30 s, after the initiation of the reaction, were measured. Black closed symbols, standard order of addition; i.e. single-stranded DNA and protein were preincubated for 10 min in (A–C) or the indicated time in (D), and the reaction was started by the addition of double-stranded DNA; blue open symbols, the reaction was initiated by the simultaneous mixing of single-stranded DNA and double-stranded DNA with wild-type or mutant RecA; red closed symbols, double-stranded DNA and protein were preincubated for 5 min in (A–C) or the indicated time in (D), and the reaction was started by the addition of single-stranded DNA. ▼, ▽ (inverted triangles), recA-D161A; ●, ○ (circles), RecA-wt. Crosses, negative control with a heterologous combination of single-stranded DNA and double-stranded DNA (pKF18K-2), under standard order of addition conditions.

We incubated radioactively labeled single-stranded DNA with a molar excess of form I DNA and sufficient amounts of RecA to obtain the formation and the following dissociation (32), with ATP and an ATP-regenerating system. After the preincubation of the ^33^P-labeled single-stranded DNA with RecA to allow nucleoprotein filament formation, the reaction was initiated by the addition of form I DNA. The generation of homologous joints was analyzed by gel electrophoresis (see the inset in Figure 6A as an example) and directly quantified by the ^33^P-signals. In the reaction with RecA-wt, the homologous joints increased to the maximum level within 1 min and then decreased (Figure 6A, black filled circles). The mutant recAs with the D161A, D161N or D161E substitution in loop L1 exhibited similar profiles of homologous joint formation and subsequent homologous joint dissociation to those of RecA-wt or recA-S162A (Supplementary Figure S5 and Figure 6).

The enhancement of homologous joint formation by the D161A substitution in loop L1 of RecA became apparent, when we compared the mutant recA and RecA-wt by changing the order of addition of protein, single-stranded DNA and double-stranded DNA to the reaction. The preincubation of RecA-wt and single-stranded DNA, before the addition of double-stranded DNA to start formation (standard order), stimulates the joint formation, as compared to the reaction started by the simultaneous addition of single-stranded and double-stranded DNAs (Figure 6A, black symbols versus blue symbols). Thus, the preincubation of RecA-family recombinases with single-stranded DNA, before the addition of double-stranded DNA, is used as the standard procedure to assay homologous joint formation. On the other hand, the preincubation of RecA-wt and double-stranded DNA, before the addition of single-stranded DNA to start the reaction, strongly suppresses the joint formation, as compared to the standard order of addition (Figure 6A, red symbols versus black symbols). As shown in Figure 6B, the D161A substitution in loop L1 of RecA removed the effects of the order of the addition.

To compare the extents of the stimulation of homologous joint formation by D161A with that by RecA-wt, we measured the homologous joint formation in the presence of various amounts of the mutant recA and RecA-wt (Supplementary Figure S6). We also varied the preincubation times of RecA with single-stranded DNA (standard order-of-addition for homologous joint-formation assay of RecA and Rad51) or with double-stranded DNA before the addition of their partner DNA. The amounts of homologous joints formed at 30 s of incubation were plotted, as representatives of the initial velocity of homologous joint formation, against the amounts of protein (Figure 6C and D). The enhancement was 3.5–6-fold when the mutant or wild-type RecA was preincubated with double-stranded DNA, 2.5-fold when both single-stranded DNA and double-stranded DNA were added simultaneously, but only 1.2-fold when RecA was incubated with single-stranded DNA before the addition of double-stranded DNA (Figure 6C and D). On the other hand, the preincubation of RecA-wt with double-stranded DNA reduced homologous joint formation by 4–6-fold, but the preincubation of recA-D161A only did so by 1.3–1.6-fold, as compared with the standard order-of-addition (Figure 6C and D). Thus, D161A reduced the suppressive effect of the protein preincubation with double-stranded DNA on homologous joint formation, but did not enhance the formation.

The stimulating and suppressing effects of the preincubation of RecA with single-stranded DNA and double-stranded DNA, respectively, on the initial velocity of homologous joint formation were time dependent. The effects of the preincubation with double-stranded DNA appeared more quickly (finished within 1 min) than the slow activation by preincubation with single-stranded DNA (more than 5 min; Figure 6D). The slow activation includes filament formation by RecA along single-stranded DNA (see (18)). The quick suppression by the preincubation with double-stranded DNA suggested that the binding of RecA to double-stranded DNA in the presence of ATP is sufficient to inactivate the RecA.

### The D161A substitution enhanced the repair of MMS-damaged DNA *in vivo*

Finally, we tested whether the alanine replacement of Asp-161 (D161A) inhibited or stimulated the repair of DNA lesions in *E. coli* cells. RecA plays two roles in DNA repair *in vivo*: first, RecA functions as a recombinase to catalyze the pairing of single-stranded regions with the homologous double-stranded DNA, which serves as a template for repair, to form homologous joints. Second, RecA promotes the cleavage of the SOS repressor, LexA, to induce the expression of SOS genes, including the *recA* gene. To exclude the possible increase in the expression level of RecA by SOS induction, we compared cells with plasmid-borne *recA*^+^ or *recA-D161A* under *tac* promoter control. These cells had the entire chromosomal deletion of *recA* (*recAΔ*) and a multi-copy plasmid bearing either the wild-type or mutant *recA*. The *E. coli* cells with *recA-D161A* did not show filamentous growth (Supplementary Figure S7), indicating that *recA-D161A* did not cause SOS constitutive. As shown in Supplementary Figure S8A, the cells with *recA-D161A* appeared more resistant to methyl MMS than the cells with *recA^+^*. A quantitative analysis more clearly showed that the cells with *recA-D161A* are more resistant to MMS than the cells with *recA^+^* (Supplementary Figure S8B). Notably, under these conditions, the SOS genes are inducible by activated RecA.

## DISCUSSION

Asp-161 in loop L1 is extremely well conserved among all but two of the 63 eubacterial RecAs examined; one of the exceptions has Glu at this position (35). Our study revealed that RecA actively selects single-stranded DNA for the primary DNA binding and that Asp-161 in loop L1 plays a crucial role in this mechanism. The D161A mutation extensively enhanced double-stranded DNA binding with only a slight effect on single-stranded DNA binding shown by the electrophoresis mobility shift assay (Figure 2), and double-stranded DNA-dependent ATP hydrolysis, by removing the effects of the presence or absence of supercoils (Figures 3 and 4A) and the stringent Mg^2+^ concentration requirements (Figure 3). These results indicated that the D161A replacement suppresses the selectivity toward single-stranded DNA in the primary DNA binding.

The selectivity for single-stranded DNA and the suppressed double-stranded DNA binding by the Asp-161 on loop L1 appeared to depend on the negative charge at this position in loop L1 (Figures 2 and 3A), and in the presence of ATP, partly on the side chain size, as indicated by a comparison of the effects of D161A and D161N substitutions (Figures 2 and 3A). The location of Asp-161 in inactive RecA appears to cause a steric barrier to the complementary strand in the activated RecA-double-stranded DNA complex (Figure 1B and C, right panel), but not with the single-stranded DNA (Figure 1B and C, left panel), in the RecA-single-stranded or double-stranded DNA complexes. In the DNA-free *Mycobacterium smegmatis* RecA (msRecA) structure (PDB IDs: 2OFO, 2OEP, 1UBG) (36–38), Asp-163 (corresponding to Asp-161 in *E. coli* RecA) also sterically clashes with the complementary strand located in the activated RecA-double-stranded DNA complex, in both the absence and presence of nucleotide cofactors (Figure 1D and E). This supports the proposal that Asp-161 of RecA provides a steric barrier and electrostatic repulsion against double-stranded DNA (but not single-stranded DNA), regardless of the presence or absence of nucleotide cofactors. Thus, using the negative charge of Asp-161, loop L1 is likely to function in the discrimination of single-stranded DNA from double-stranded DNA in the primary DNA-binding step.

The primary DNA binding induces the hydrolysis of ATP and LexA-repressor cleavage. The enhancement of double-stranded DNA binding by the D161A replacement (Figure 2) correlated well with the enhancement of the double-stranded DNA-dependent ATPase activity of recA-D161A (Figures 3 and 4) and double-stranded DNA-dependent LexA cleavage (Figure 5). These results further support the proposal that Asp-161 in loop L1 governs the single-stranded DNA specificity in the primary DNA-binding step.

The selectivity for single-stranded DNA in the primary binding step of RecA appears to enable RecA targeting to double-strand break sites to be repaired, since the resection from a break end yields a single-stranded tail, which is unique to the break sites. Then, one may suppose that the lower selectivity of RecA by the D161A replacement would decrease the DNA repair efficiency. The *E. coli* mutant bearing *recA-D161N* was previously classified as *recA^+^* (39). In contrast to the above expectation, we found that the *E. coli* cells expressing recA-D161A exhibited significantly increased repair capacity toward DNA damage by MMS (Supplementary Figure S8). To understand these observations, we need to consider the recombination mediators required by RecA, and the SOS-mediated DNA repair functions in *E. coli* cells.

In cells, RecA requires a recombination mediator (RecOR or RecBCD) to bind to single-stranded DNA regions, where SSB binds first. The single-stranded regions are recognized by SSB in *E. coli* cells, and the recombination mediator recruits RecA to the SSB-bound single-stranded region. Thus, the preference of RecA for single-stranded DNA is not essential to initiate recombination at the single-stranded region.

In the *E. coli* cells used in this study, the original *recA^+^* gene was removed, and the mutant and wild-type *recAs* to be tested were solely expressed under the *tac* promoter on a multiple copy-number plasmid (pKK223-3), with a pBR322-derived replication origin (40). Although this system excludes the effects of RecA overproduction by SOS induction, the cells are likely to have an excess of RecA protein, and recA-D161A is more active in LexA repressor cleavage (Figure 5). Thus, the excess of recA-D161A would increase the expression of SOS-inducible DNA repair genes, providing recA-D161A-expressing cells with higher repair capacity. Although the D161A substitution allows RecA to use double-stranded DNA as a cofactor for the LexA-cleavage activity, the *E. coli* cells with *recA-D161A* were normal and did not show filamentous growth (Supplementary Figure S7), indicating the absence of spontaneous SOS induction. This observation suggests that the enhanced double-stranded DNA-binding capacity of recA-D161A by itself is not sufficient for SOS induction *in vivo*.

**Figure 7.**
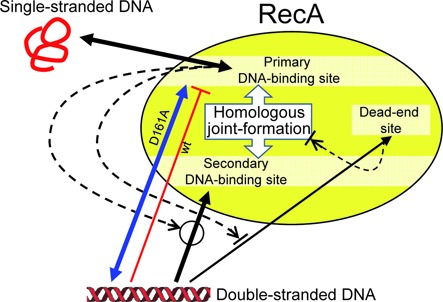
Model that explains the D161A-substitution-mediated reduced suppression of homologous joint formation in preincubation reaction of RecA with double-stranded DNA before interacting with single-stranded DNA. RecA, either wild-type or D161A mutant, in ATP-bound form first binds single-stranded DNA at its primary DNA-binding site, resulting in activation of the secondary DNA-binding site (‘gateway’) for double-stranded DNA bind (to form a ternary complex of RecA, single-stranded DNA and double-stranded DNA), followed by homologous joint formation. If RecA-wt interacts with double-stranded DNA before interacting with single-stranded DNA, RecA and the double-stranded DNA form a dead-end binary complex (at an assumed ‘dead-end site’), resulting in elimination of the RecA-wt from homologous joint formation. On the other hand, recA-D161A interacts with double-stranded DNA before interacting with single-stranded DNA, the double-stranded DNA preferentially binds to the primary DNA-binding site, which induces ATP hydrolysis, resulting in the release of the double-stranded DNA and making the recA-D161A available to homologous joint formation. The dead-end site may be the gateway of single-stranded DNA-free RecA, or another site than the primary and the secondary DNA-binding site. 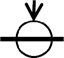, a process that requires the indicated signal; 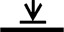, a process that is inhibited by the indicated signal. Arrows, processes and their direction; |—, process not allowed.

The most prominent effect of the D161 replacement on homologous joint formation is the reduced suppression in the preincubation reaction of recA-D161A with double-stranded DNA before the addition of single-stranded DNA, as shown by the order-of-addition experiments (Figure 6). Inverse homologous joint formation (41), which is joint formation by the ATP-activated recA-D161A-double-stranded DNA filamentous complex and free single-stranded DNA, may explain the reduced suppressive effects of the preincubation of the mutant protein with double-stranded DNA. However, we have not obtained clear experimental support for this possibility, and thus, we would like to propose another explanation (Figure 7). To explain the reduced suppression of homologous joint formation by the preincubation with double-stranded DNA of recA-D161A, we should consider the presence of at least two distinct DNA-binding sites (or surfaces) on RecA and Rad51; i.e. one facing the inside of the RecA/Rad51 filaments for the primary binding that activates ATP hydrolysis (22) and the other for binding double-stranded DNA to ATP-activated RecA-single-stranded DNA filaments (characterized as the secondary DNA-binding site or a ‘gateway’; 15,42). Our results suggested that the binding of the DNA-free ATP-bound form of RecA to double-stranded DNA has two modes: one that activates RecA for ATP hydrolysis through binding to the primary binding site and the other that inactivates RecA, as for homologous joint formation, through dead-end binary product formation. The DNA-binding site for the dead-end product formation (‘dead-end site’) may overlap with the gateway for the DNA-free RecA (and Rad51) or another place on RecA. The results of the order-of-addition experiments are explained as follows: for the DNA-free wild-type RecA, double-stranded DNA can bind only to the dead-end site (and dissociates from the site slowly, if at all), and the double-stranded DNA-bound RecA is eliminated from the homologous joint formation. On the other hand, double-stranded DNA can bind to DNA-free recA-D161A through either the primary site or the dead-end site, but the binding through the primary site is reversible because the binding triggers ATP hydrolysis by RecA, which stimulates the release of the double-stranded DNA from the RecA. The slight suppression by the preincubation of recA-D161A suggests that double-stranded DNA prefers the primary site of the mutant recA, rather than the dead-end site. Examination of the effects of the order of addition experiments among RecA, single-stranded DNA and double-stranded DNA on the ternary complex (a recombinase-reaction intermediate: see the Introduction section and Figure 7; 17–19) formation, and the effects of amino acid-replacements in the gateway on homologous joint formation by RecA-wt and recA-D161A would be tests for the model shown in Figure 7 and possible inverse homologous joint formation by recA-D161A.

The incubation of RecA/Rad51 recombinases with double-stranded DNA, before the addition of single-stranded DNA, is known to prevent homologous joint formation by these recombinases (see Figure 6A and (43) as examples of *E. coli* RecA and yeast Rad51, respectively). This study revealed that this inhibitory role of the contact of RecA with double-stranded DNA, before the binding to single-stranded DNA, is actively regulated by Asp-161 on loop L1, and may have a biological function, which is under investigation.

## ACCESSION NUMBER

The structural coordinates of the DNA-free inactive RecA crystal were deposited in the Protein Data Bank, under the accession code 4TWZ.

## SUPPLEMENTARY DATA

Supplementary Data are available at NAR Online.

SUPPLEMENTARY DATA
