# Supplementary material for: Loop L1 governs the DNA-binding specificity and order for RecA-catalyzed reactions in homologous recombination and DNA repair
Source: Nucleic Acids Res. 2015 Jan 5;43(2):973–86. doi: 10.1093/nar/gku1364 (PMC4333409; doi:10.1093/nar/gku1364)
Supplement: SUPPLEMENTARY DATA [file supp_43_2_973__index.html]

Loop L1 governs the DNA-binding specificity and order for RecA-catalyzed reactions in homologous recombination and DNA repair — Loop L1 governs the DNA-binding specificity and order for RecA-catalyzed reactions in homologous recombination and DNA repair — SUPPLEMENTARY DATA 

# Loop L1 governs the DNA-binding specificity and order for RecA-catalyzed reactions in homologous recombination and DNA repair

## SUPPLEMENTARY DATA

**Files in this Data Supplement:**

- SUPPLEMENTARY DATA
