# Supplementary material for: Loop L1 governs the DNA-binding specificity and order for RecA-catalyzed reactions in homologous recombination and DNA repair
Source: Nucleic Acids Res. 2015 Jan 5;43(2):973–86. doi: 10.1093/nar/gku1364 (PMC4333409; doi:10.1093/nar/gku1364)
Supplement: SUPPLEMENTARY DATA [file supp_gku1364_nar-01820-d-2014-File003.pdf]

Supplementary Data

# Loop L1 governs the DNA-binding specificity and order for RecA-catalyzed reactions in homologous recombination and DNA repair

Takeshi Shinohara[1,2,3], Shukuko Ikawa[1,2], Wakana Iwasaki[1], Toshiki Hiraki[4], Takaaki Hikima[4], Tsutomu Mikawa[1], Satoko Akashi[3], Naoto Arai[5], Nobuo Kamiya[4] and Takehiko Shibata[1,2,3 *]

[1] Cellular & Molecular Biology Unit, RIKEN, Wako-shi, Saitama 351-0198, Japan
[2] Advanced Catalysis Research Group, RIKEN Center for Sustainable Resource Science, Wako-shi, Saitama 351-0198, Japan
[3] Department of Supramolecular Biology, Graduate School of Nanobiosciences, Yokohama City University, 1-7-29 Suehiro-cho, Tsurumi-ku, Yokohama, Kanagawa 230-0045, Japan
[4] Advanced Photon Technology Division, Research Infrastructure Group, RIKEN SPring-8 Center, RIKEN Harima Institute, 1-1-1 Kouto, Sayo, Hyogo 679-5148, Japan
[5] Department of Applied Biological Science, Nihon University College of Bioresource Sciences, Fujisawa-shi, Kanagawa 252-0880, Japan

### Crystal structure of free RecA

Crystals typically grew within a week to a maximum size of approximately 300 x 50 x 20 μm. Electrophoresis of the crystals revealed a single band corresponding to RecA, with no signal for ARM193Fab (see Supplemental Materials and Methods). The crystals belonged to the $P6_1$ space group, with unit cell dimensions of $a = b = 103.7$ Å and $c = 72.1$ Å. One monomer of the RecA protein without the Fab fragment was found in the asymmetric unit, and the solvent content was calculated to be 58 %. If we assumed that both RecA and the Fab fragment were present in the asymmetric unit, then the solvent volume would be less than 10 %. Higher amounts of the Fab fragment of the ARM193 antibody were required to obtain complete inhibition of some RecA activities (three molecules of ARN 193Fab per one molecule of the RecA protomer: 44), suggesting that an insufficient amount of the Fab fragment was used for co-crystallization with RecA.

Residues 1-4 and 332-352 were disordered. The crystal data and the statistics for data collection and structure determination are summarized in Supplementary **Table S1**. The structural coordinates were deposited in the Protein Data Bank, under the accession code 4TWZ. The final refined model included residues 6 to 332 out of the 352 total protein residues, and showed that 95.6% of the residues were in the favored region of the Ramachandran plot, and the rest were in the allowed region (45). Part of loop L2, residues 200-203, was in an area of weak electron density and was not defined clearly. The $F_o$-$F_c$ electron density omit map of loops L1 and L2 is shown in **Fig. 1A**.

The present crystal had a filament pitch of 72.1 Å, and this helical pitch is smaller than that in the first crystal structure of free RecA reported by Seitz's group (82.7 Å) (21), and similar to that of the protomers forming the "compressed" filament reported by Xing and Bell (approximately 74 Å) (46). Since this pitch size is similar to that of the inactive filament observed previously by small-angle neutron scattering (47) and electron microscopy (48,49), the crystallographic filament in the obtained

free RecA crystals is considered to reflect an inactive state. The structure of the monomer and its packing arrangements are essentially identical to those of the previously reported 74 Å filaments (46, r.m.s.d. of 0.57-0.73 Å for Cαs in a monomer) except for loops L1 and L2, which are described in the main text. Our inactive RecA crystal provided an electron density map for most of both loops L1 and L2, unlike previously reported DNA-free RecA crystals (**Fig. 1A**). In the DNA-free RecA crystal structures reported to date, the electron densities for loops L1 and L2 are highly ambiguous, and the structures of these loops have been provided for four RecA structures: the free RecAs from *E. coli* (this study), and the RecAs from *Mycobacterium smegmatis* (msRecA) in the free (PDB ID: 2OFO), ADP-bound (PDB ID: 2OEP), and dATP-bound (PDB ID: 1UBG) forms (36-38).

The configuration of the protomers in our putative inactive filament distinctly differs from that in the activated RecA-DNA complex forming extended filaments, in which the helical pitches range between 92.4 - 95.3 Å (22, Supplementary **Fig. S1A**, right panel). The core and the C-terminal domains in each protomer of this inactive RecA and those of the activated RecA-DNA complexes (22) superimposed well (within an rmsd. of 1.03 Å for Cαs of residues 45-156, 165-194 and 210-328), but the orientations of the N-terminal domains and the conformations of loops L1 and L2 significantly differed. The N-terminal domain (residues 1-33), containing an α-helix and a β-strand, is reportedly essential for RecA polymerization (50,51). In spite of the large difference in the configurations between the central core domains, the interprotomer interactions via the N-terminal domain were conserved in both the active and inactive filaments (Supplementary **Fig. S1B**). The flexible linker region modulates the location of the N-terminal domain, to retain the interprotomer contacts responding to the movement of the central core domains. The interaction surface area, excluding the N-terminal domain, was 684.0 $Å^2$ in this putative inactive filament, which is less than half of that in the active filament, ~1,460$Å^2$. In the active filament, the hydrogen bonds formed between the γ-phosphate of ATP and K248 and K250 of the adjacent molecule are assumed to be critical to stabilize the active RecA-RecA interface (22) (Supplementary **Fig. S1C**). In the inactive free RecA filament, the putative catalytic residue Glu96 is shielded from the solvent by loop L2 of the adjacent protomer (Supplementary **Fig. S1D**).

## Supplemental Materials and Methods

### Preparation of DNA for biochemical analysis

The 90-nucleotide single-stranded DNA (10 μM in polynucleotide, 900 μM in nucleotides) was labeled with $^{33}P$ at the 5' terminus by an incubation in reaction buffer (10 μL) with [γ-$^{33}$P]ATP and 1U/μL T4 polynucleotide kinase at 37°C for 30 min, as recommended by the manufacturer (Megalabel DNA 5' end labeling kit, Takara Bio Inc.). The preparation was heated at 90°C for 2 min to inactivate the kinase, and the unreacted ATP was removed by two rounds of spin column separation. The labeled single-stranded DNA was diluted 80-fold in TE buffer (10 mM Tris-HCl, pH 7.5, and 0.1 mM EDTA). The amounts of the labeled single-stranded DNA indicated are the calculated values, assuming 100% recovery during the labeling process.

Negatively supercoiled closed circular double-stranded (form I) pBluescript SK(-) DNA and M13 circular single-stranded DNA were prepared by the procedures described previously (25,26). pGsat4 form I DNA was described previously (52). ΦX174 circular single-stranded DNA was purchased from New England BioLabs, Inc. (Ipswich, MA, USA). It is noted that the form I DNA prepared by procedures including alkaline-treatment often traps non-canonical structures, and thus should not be used in the assay for homologous joint-formation.

Poly(dT) was prepared by incubating 10 pmol (in molecules)/μL oligo$(dT)_{30}$ and 1.6 mM dTTP with terminal deoxynucleotidyl transferase (0.3 U/μL, purchased from Takara Bio Inc., Shiga, Japan) in a reaction mixture (50 μL, consisting of 100 mM HEPES buffer (pH 7.2), 8 mM $MgCl_2$, 0.1 mM dithiothreitol, 0.02% bovine serum albumin) at 32°C for 5 hr. After the reaction was terminated, poly(dT) was purified by phenol-chloroform extraction followed by ethanol precipitation. The average chain length of poly(dT) was more than 100 nucleotides.

**DNA constructs for mutant recA expression**

The site-directed mutagenesis of *recA* was performed as described previously (15), with a slight modification.

**Purification of RecA-wt and mutant recAs**

The mutant recAs and RecA-wt were expressed from a derivative of the multicopy vector pKK223-3, which carried the mutant *recA* or *recA*$^+$ under the control of the *tac* promoter in a *ΔrecA* derivative of *E. coli*. All RecAs were purified by the procedures described previously (25,26), with the following modifications. After ammonium sulfate precipitation, the proteins were fractionated by TOYOPEARL butyl-650M (TOSOH) column chromatography with a linear gradient of ammonium sulfate (1 M to 0 M), followed by hydroxyapatite (CHT-1, BioRad) column chromatography.

It is noted that we used fresh preparations of the mutant recAs and RecA-wt purified from freshly prepared cell-lysates obtained from newly transformed *E. coli* cells.

We experienced some experimental difficulties when we used the wild type RecA purified from old 60% glycerol stock of partially purified RecA in a freezer, which reproducibly showed very slow homologous joint-formation without the following dissociation phase (Supplementary **Fig. S9A**). We found that the RecA preparation (called RecA*) contained a higher amounts of mono- and di-oxidized forms, as revealed by mass-spectrometry performed (Supplementary **Fig. S9C**).

**Fab fragment of the ARM193 antibody**

The ARM193 antibody (approximately 4 mg/ml, purchased from Medical & Biological Laboratories Co.,Ltd (MBL)) was treated with papain, as described (44), and the Fab product was purified by DEAE-5PW (TOSOH) chromatography followed by Mono-S (Pharmacia) chromatography. The purified Fab fragment was tested for the ability to prevent RecA-filament formation, by a gel filtration assay on TSK-GEL G4000SW resin (TOSOH) (See ref. 44).

**Crystallization**

Prior to the preparation of crystallization droplets, RecA was mixed with the Fab fragments of the ARM193 antibody (ARM193Fab) at a 1:1 molar ratio at 277 K, to produce a protein solution including 1.9 mg/ml RecA protein and 2.5 mg/ml ARM193Fab, in 10 mM MES (pH 6.5) buffer. The crystallization droplets were prepared by mixing 10 μL of the protein solution and 10 μL of reservoir solution, which contained 24 % PEG400, 10 % glycerol, 10 mM $MgCl_2$ and 0.1 M MES, pH 6.5. The crystals were grown by the hanging drop vapor diffusion method at 293 K. The ARM193Fab promotes the dissociation of spontaneously formed RecA filaments into monomers (44), and we expected that the addition of the ARM193Fab would improve the crystallization of RecA. However, the Fab was not included in the crystal that we obtained (see Supplementary **Fig. S1**).

The RecA crystal was briefly soaked in a cryo-protectant solution, containing 34 % PEG400, 18 % glycerol, 10 mM $MgCl_2$ and 0.1 M MES (pH 6.5), and then flash cooled in a stream of $N_2$ gas at 100 K. X-ray diffraction data were collected at the RIKEN beamline II (BL44B2) at SPring-8 in Harima, Japan (53). The diffraction images were recorded on a CCD detector (MAR165), and were processed using the HKL2000 software package (54) up to 2.8 Å resolution. The initial phase was derived by molecular replacement with the program MOLREP in CCP4 (55), using the atomic coordinates of the *E. coli* RecA protein (PDB ID: 2REB) previously reported by Steitz's group (21) as a probe. The molecular structure was constructed and modified using TURBO FRODO (56), and refined using CNS (57).

**Nano-electrospray mass spectrometry**

Nano-electrospray mass spectra were acquired on a Waters (Manchester, UK) SYNAPT G2 quadrupole ion mobility time-of-flight mass spectrometer (see refs. 58,59). A 4 μL aliquot of 16 μM RecA in 50 mM ammonium acetate was deposited in a nanospray tip with an 8-μm internal diameter (HUMANIX, Hiroshima, Japan) and placed in a nanospray ion source. A capillary voltage of 0.8–0.9 kV and a sample cone voltage of 20–30 V were typically applied. Each mass spectrum was acquired in 1 s, and more than 100 mass spectra were accumulated and smoothed by the Savitzky-Golay method. All data analysis and processing were performed with MassLynx v4.1 (Waters).

**Supplementary References**


44. Ikawa, S., Kamiya, N. and Shibata, T. (1989) Defective homologous pairing and proficient processive unwinding by the *recA430* mutant protein and intermediates of homologous pairing by recA protein. J. Biol. Chem., 264, 21167-21176.
45. Chen, V.B., Arendall, W.B., 3rd, Headd, J.J., Keedy, D.A., Immormino, R.M., Kapral, G.J., Murray, L.W., Richardson, J.S. and Richardson, D.C. (2010) MolProbity: all-atom structure validation for macromolecular crystallography. Acta Crystallogr D Biol Crystallogr, 66, 12-21.
46. Xing, X. and Bell, C.E. (2004) Crystal structures of Escherichia coli RecA in a compressed helical filament. J. Mol. Biol., 342, 1471-1485.

47. DiCapua, E., Schnarr, M., Ruigrok, R.W., Lindner, P. and Timmins, P.A. (1990) Complexes of RecA protein in solution. A study by small angle neutron scattering. J. Mol. Biol., 214, 557-570.
48. Yu, X. and Egelman, E.H. (1992) Structural data suggest that the active and inactive forms of the RecA filament are not simply interconvertible. J. Mol. Biol., 227, 334-346.
49. Ruigrok, R.W.H., Bohrmann, B., Hewat, E., Engel, A., Kellenberger, E. and Dicapua, E. (1993) The inactive form of RecA protein: the compact structure. EMBO J., 12, 9-16.
50. Mikawa, T., Masui, R., Ogawa, T., Ogawa, H. and Kuramitsu, S. (1995) N-terminal 33 amino acid residues of *Escherichia coli* RecA protein contribute to its self-assembly. J. Mol. Biol., 250, 471-483.
51. Masui, R., Mikawa, T. and Kuramitsu, S. (1997) Local folding of the N-terminal domain of Escherichia coli RecA controls protein-protein interaction. J. Biol. Chem., 272, 27707-27715.
52. Kagawa, W., Kurumizaka, H., Ikawa, S., Yokoyama, S. and Shibata, T. (2001) Homologous pairing promoted by the human Rad52 protein. J. Biol. Chem., 276, 35201-35208.
53. Adachi, S., Oguchi, T., Tanida, H., Park, S.-Y., Shimizu, H., Miyatake, H., Kamiya, N., Shiro, Y., Inoue, Y., Ueki, T. *et al.* (2001) The RIKEN structural biology beamline II (BL44B2) at the SPring-8. Nucl. Instrum. Methods Phys. Res., A, 467–468, 711-714.
54. Otwinowski, Z. and Minor, W. (1997) In Carter, C. W. J. and Sweet, R. M. (eds.), Macromolecular Crystallography, part A. Academic Press, New York, Vol. 276, pp. 307-326.
55. Collaborative Computational Project, N. (1994) The CCP4 Suite: Programs for Protein Crystallography. Acta Cryst., D, 50, 760-763
56. Jones, T.A. (1978) A graphics model building and refinement system for macromolecules. J Appl Cryst, 11, 268-272.
57. Brunger, A.T., Adams, P.D., Clore, G.M., DeLano, W.L., Gros, P., Grosse-Kunstleve, R.W., Jiang, J.-S., Kuszewski, J., Nilges, M., Pannu, N.S. *et al.* (1998) Crystallography & NMR System: A New Software Suite for Macromolecular Structure Determination. Acta Cryst, D, 54, 905-921.
58. Saikusa, K., Fuchigami, S., Takahashi, K., Asano, Y., Nagadoi, A., Tachiwana, H., Kurumizaka, H., Ikeguchi, M., Nishimura, Y. and Akashi, S. (2013) Gas-phase structure of the histone multimers characterized by ion mobility mass spectrometry and molecular dynamics simulation. Anal. Chem., 85, 4165-4171.
59. Saikusa, K., Kuwabara, N., Kokabu, Y., Inoue, Y., Sato, M., Iwasaki, H., Shimizu, T., Ikeguchi, M. and Akashi, S. (2013) Characterisation of an intrinsically disordered protein complex of Swi5-Sfr1 by ion mobility mass spectrometry and small-angle X-ray scattering. Analyst, 138, 1441-1449.

## Supplemental figure legends

### Figure S1. The RecA crystal.

(**A**) Comparison of the protomer configuration in the filaments observed in the inactive RecA crystal structure that we determined (left), and those in the activated RecA-single-stranded DNA complex (22, right). Although both filaments have six protomers per helical turn, only three protomers are shown for clarity. The pink-colored protomers in the two filaments are shown in the same orientation, and the configurations of the two adjacent protomers, colored cyan and green, are compared. The residues containing the interprotomer contacting atoms (within 4 Å) are highlighted in deep colors.
(**B**) The interprotomer interactions involving the N-terminal α-β motif (colored green, encircled by red dashed lines) are conserved in both the inactive (left) and active (right) filaments.
(**C**) Protomer-protomer interfaces in the filaments of inactive RecA (white and grey) and the activated RecA-single-stranded DNA complex (22, magenta and green). The ADP-$AlF_4$-$Mg^{2+}$ and the single-stranded DNA in the activated filament are colored red and blue, respectively. K248 and K250, which coordinate the $AlF_4$ in the activated filament, are depicted by black (inactive) and green (active) cpk models. The white and magenta protomers are superimposed, and the orientations of the adjacent protomers (green and grey) are compared. The yellow arrow indicates the movement of the protomers, from the inactive state (grey) to the active state (green).
(**D**) Magnified view of the protomer-protomer interface of inactive RecA, colored as in panel A. The putative catalytic residue E96 (red) and the residues involved in interprotomer hydrogen bonds are shown in stick representations. All molecular graphics images were generated using Pymol (http://www.pymol.org).

### Figure S2. Calculation of the RecA concentrations required to achieve half-maximum binding shown in Fig. 2E

The values obtained are 0.54 μM for recA-D161A and recA-D161N, and 0.64 μM for RecA-wt in the presence of ATP. Those in the absence of ATP, 0.29 μM for recA-D161A and 0.47 μM for recA-D161N and RecA-wt. The differences in the calculated percentages of RecA-bound 90-nucleotide single-stranded DNA among mutants and wild-type RecAs were mostly within error bars.

### Figure S3. Amino acid-replacement of Asp-161 by Ala (D161A) or Asn (D161N) in loop L1 enhances double-stranded DNA-binding, but not single-stranded DNA-binding under the 1.0 mM $MgCl_2$ conditions.

**A** and **B**. Single-stranded DNA-binding.

90-nucleotide single-stranded DNA (5.0 μM) labelled at 5' terminus with FAM (6-carboxyfluorescein) was incubated in the standard buffer except with 1.0 mM $MgCl_2$ with the indicated amounts of mutant or wild-type RecA for 5 min in the presence (A) or absence (B) of ATP. The samples were then fixed and subjected to agarose gel electrophoresis. The fluorescence in the gel was analyzed.

Amounts of RecA in A and B: lanes 1 and 16, no RecA; lanes 2, 9 and 17, 0.05 μM; lanes 3, 10

and 18, 0.1 μM; lanes 4, 11 and 19, 0.25 μM; lanes 5, 12 and 20, 0.5 μM; lanes 6, 13 and 21, 1.0 μM; lanes 7, 14 and 22, 2.0 μM; lanes 8, 15 and 23, 3.0 μM.

Arrows i, unbound single-stranded DNA; ii and iii, RecA bound to single-stranded DNA.

**C** and **D**, Double-stranded DNA-binding

Unlabeled pBluescript SK(-) supercoiled (form **I**) double-stranded DNA (18 μM) was incubated in the standard buffer except with 1.0 mM $MgCl_2$ for 15 min with the indicated amounts of mutant or wild-type RecA in the presence (C) or absence (D) of ATP. The samples were then subjected to agarose gel electrophoresis without fixation. The gel was stained with ethidium bromide and the DNA signals were detected by UV irradiation. Contrast was enhanced.

Amounts of RecA are: lanes 1 and 16, no RecA; lanes 2, 9 and 17, 0.5 μM; lanes 3, 10 and 18, 1.0 μM; lanes 4, 11 and 19, 2.0 μM; Lanes 5, 12 and 20, 3.0 μM; lanes 6, 13 and 21, 4.0 μM; lanes 7, 14 and 22, 5.0 μM; lanes 8, 15 and 23, 10 μM. Lane M, DNA size markers. Contrast was enhanced.

Arrows i, free form **I**; ii, nicked circular double-stranded DNA present in the form **I** preparation; iii, RecA-bound double-stranded DNA.

**Figure S4. Effects of amino acid-replacements for Asp-161 in loop L1 on the single-stranded DNA-dependent ATPase activity of RecA.**

[$^{14}$C]ATP (26 nmol at 1.3 mM) was incubated with the indicated amounts of mutant recA or RecA-wt in the presence of 10 μM M13 single-stranded DNA for 15 min. The reaction products were fractionated by thin layer chromatography, and the amounts of ATP hydrolyzed were determined by analyzing the $^{14}$C-signals. The mutant recAs used in these experiments are indicated within each panel. Each point indicates the average obtained from four to two independent experiments using RecA-wt and mutant recAs, respectively.

**Figure S5. The formation and subsequent branch-migration of homologous joints by various mutant recAs.**

The 90-nucleotide single-stranded [$^{32}$P]DNA (0.3 μM; these experiments only) and a mutant or wild type RecA (2.0 μM) were first incubated for 10 min, and the reaction was started by the addition of homologous (pBluescript SK(-)) closed circular double-stranded DNA (18 μM). After incubations for the indicated times, aliquots were withdrawn and the reaction was terminated. Unlike the standard conditions, the proteins were removed from the samples by a treatment with Proteinase K at 37°C, and then the reaction products were analyzed by agarose-gel electrophoresis. $^{32}$P-signals were analyzed quantitatively. Mutant recAs used in these experiments are indicated within the panel. Each point indicates the average obtained from two to three independent experiments.

**Figure S6. The formation and subsequent branch-migration of homologous joints by various amounts of RecA-wt and recA-D161A, and the effects of the addition order of the DNA substrate and protein.**

**A**. The reactions with RecA-wt. **B**. The reactions with recA-D161A.

In the standard order of addition, the 90-nucleotide single-stranded [$^{33}$P]DNA (final 0.05 μM) and the indicated amounts of RecA-wt or recA-D161A were incubated for 5 min or more (preincubation), and the reaction was then started by the addition of homologous (pBluescript SK(-)) form I (negatively supercoiled closed circular double-stranded) DNA (18 μM). After incubations for the indicated times, aliquots were withdrawn and the amounts of homologous joints formed were measured, as described in Fig. 6.

Black symbols, standard order of addition of protein, single-stranded DNA and double-stranded DNA. Red symbols, double-stranded DNA was preincubated with RecA, and the reaction was then started by the addition of single-stranded [$^{33}$P]DNA. The protein amounts are indicated within the panels.

**Figure S7. D161A-replacement does not cause filamentous cell growth**

If SOS-functions are induced in *E. coli* cells, then cell division is blocked and the cells grow into long filaments. The MV1184 cells (*recAΔ*) with a multi-copy plasmid harboring *recA*$^{+}$ or *recA-D161A* under the *tac* promoter, were grown to logarithmic phase, and observed under a microscope. The microscopic images were photographed and their contrast was enhanced. Size standards are indicated in each panel. A. Empty vector (pKK223-3); B. *recA*$^{+}$ on pKK223-3; C. *recA-D161A* on pKK223-3.

**Figure S8. D161A-replacement in loop L1 confers resistance to MMS in *E. coli* cells.**

**A**. *E. coli* MV1184 (*recAΔ*) cells with a pKK223-3 plasmid bearing *recA-D161A* or *recA-wt* under the control of the *tac* promoter, or with the empty plasmid, were grown and diluted with M9 buffer to an $OD_{600nm}$ of 1.0. The cell cultures were diluted as indicated, and spotted on LB agar plates containing ampicillin, tetracycline and the indicated amounts of methyl methanesulfonate (MMS). The spotted plates were incubated at 37°C for 22 h (on plates without MMS), 24 h (on 7.0 mM MMS-containing plates) or 48 h (on 9.3 and 11.7 mM MMS-containing plates). The photos are representative of two independent experiments.

**B**. The cultures were diluted to appropriate colony counts, plated on LB agar media containing ampicillin, tetracycline and the indicated amounts of MMS, and incubated at 37°C for 1 day (on plates without MMS and on 1.7-3.5 mM MMS-containing plates for *recA-wt* or *recA-D161A* expressing strains) or 2 days (on 0.58-1.7 mM MMS-containing plates for the strain bearing the vector without *recA* and 7-11.7 mM MMS-containing plates for the *recA*$^{+}$ or *recA-D161A* expressing strains). The numbers of colonies formed on each plate were counted. The results of two independent experiments were plotted. The genotypes of *recA* on the plasmids: ▲ (triangles), *recA-D161A*; ● (circles), *recA*$^{+}$; ■ (squares), empty vector.

**Figure S9. Abnormal homologous joint-formation by a preparation of RecA-wt (RecA*) purified from an old partially purified RecA stock in a freezer**

The preparation of RecA* (see Purification of RecA-wt and mutant recAs in Supplemental Materials

and Methods) showed a slow homologous joint-formation without the following dissociation of homologous joint, even when excess protein was present.

**A**. Abnormal homologous joint formation by RecA* under the standard conditions.
The 90-nucleotide single-stranded [$^{33}$P]DNA (final 0.05 μM) and 2.0 μM RecA* or RecA-wt were incubated for 5 min or longer (preincubation), and then the reaction was initiated by the addition of 18 μM homologous (pBluescript SK(-)) form I DNA (the standard order of addition), incubated and analyzed as described in Fig 6.

▼ (inverted triangles), RecA*; ● (circles), normal RecA-wt (the data shown in Fig. 6A)

**B** and **C**. Nanospray ESI-TOF MS of RecA preparations
Analyses of a normal RecA-wt preparation (B) and an abnormal RecA* preparation (C) revealed that the RecA* preparation contained RecA variants with molecular masses that were approximately larger than RecA-wt by multiples of the mass of oxygen. The amounts of the mono-oxidized and di-oxidized RecAs in the RecA* preparation is significantly larger (*ca.* 56%) than those in the normal RecA-wt preparation (*ca.* 36%). Thus, the oxidized RecAs would cause slower homologous joint-formation without dissociation. The oxidization may have biological significance, which we have not identified.

**Table S1. Crystallographic data collection and refinement statistics**

| | |
|---|---|
| **Data collection** | |
| Space group | $P6_1$ |
| Cell dimensions | |
| *a*, *b*, *c* (Å) | 103.7, 103.7, 72.1 |
| Resolution (Å) | 20-2.8 (2.9-2.8)* |
| $R_{sym}$ | 0.094 (0.368)* |
| $I / \sigma I$ | 25.5 (5.8)* |
| Completeness (%) | 100 (100)* |
| Redundancy | 11.4 (11.4)* |
| | |
| **Refinement** | |
| Resolution (Å) | 19.8 – 2.8 |
| No. reflections | 10961 |
| $R_{work}$ / $R_{free}$ | 0.168 / 0.227 |
| No. atoms | |
| Protein | 2302 |
| $Mg^{2+}$ | 1 |
| Water | 115 |
| *B*-factors | |
| Protein | 37.7 |
| $Mg^{2+}$ | 18.1 |
| Water | 34.8 |
| Root-mean-square deviations | |
| Bond lengths (Å) | 0.012 |
| Bond angles (°) | 1.7 |

*Values in parentheses are for highest-resolution shell.

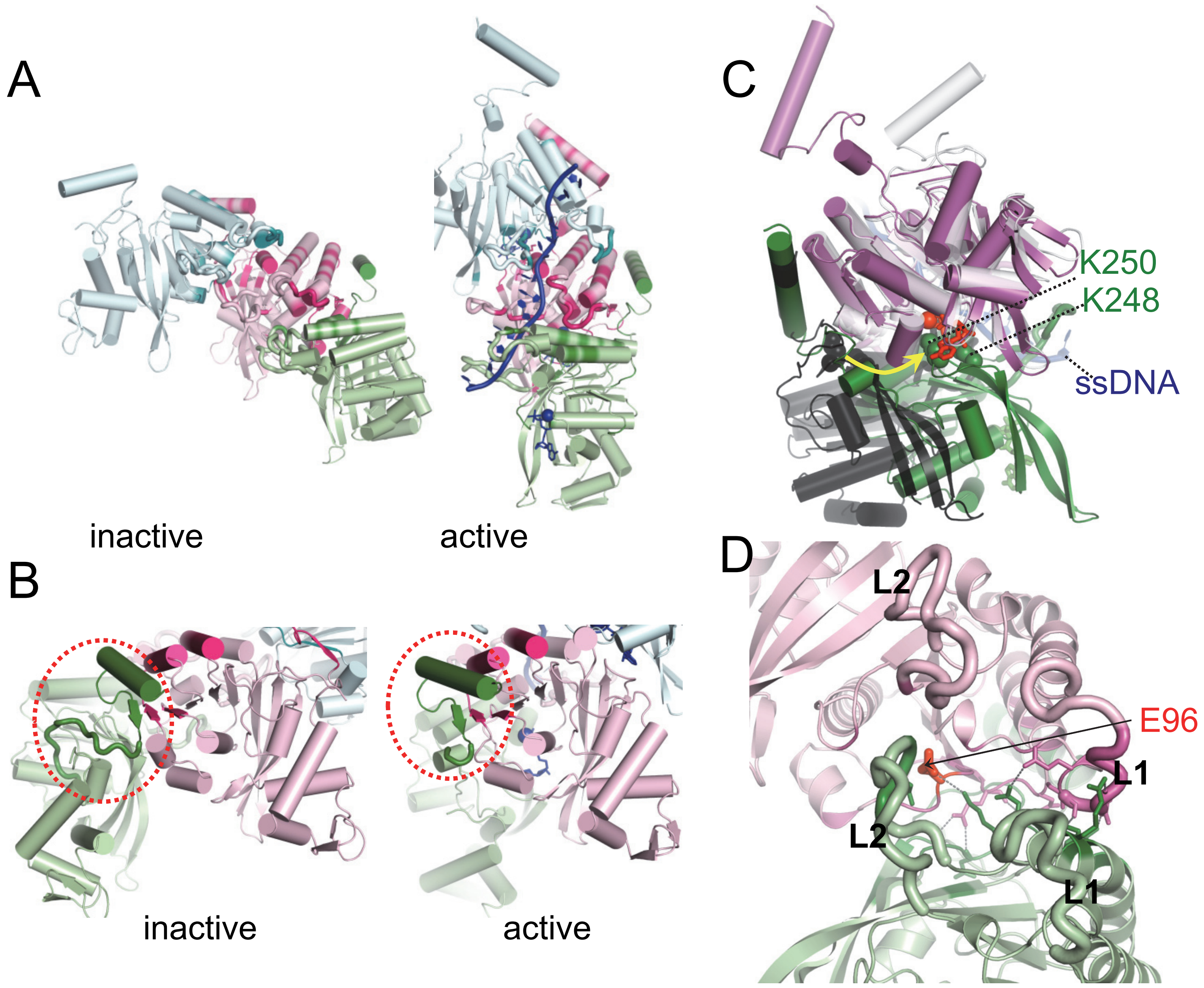

A
inactive
active
B
inactive
active
C
K250
K248
ssDNA
D
L2
E96
L1
L2
L1


Figure S1

# [RecA] for a half-maximum binding from Fig. 2E
## ssDNA, +ATP, 13 mM $MgCl_2$

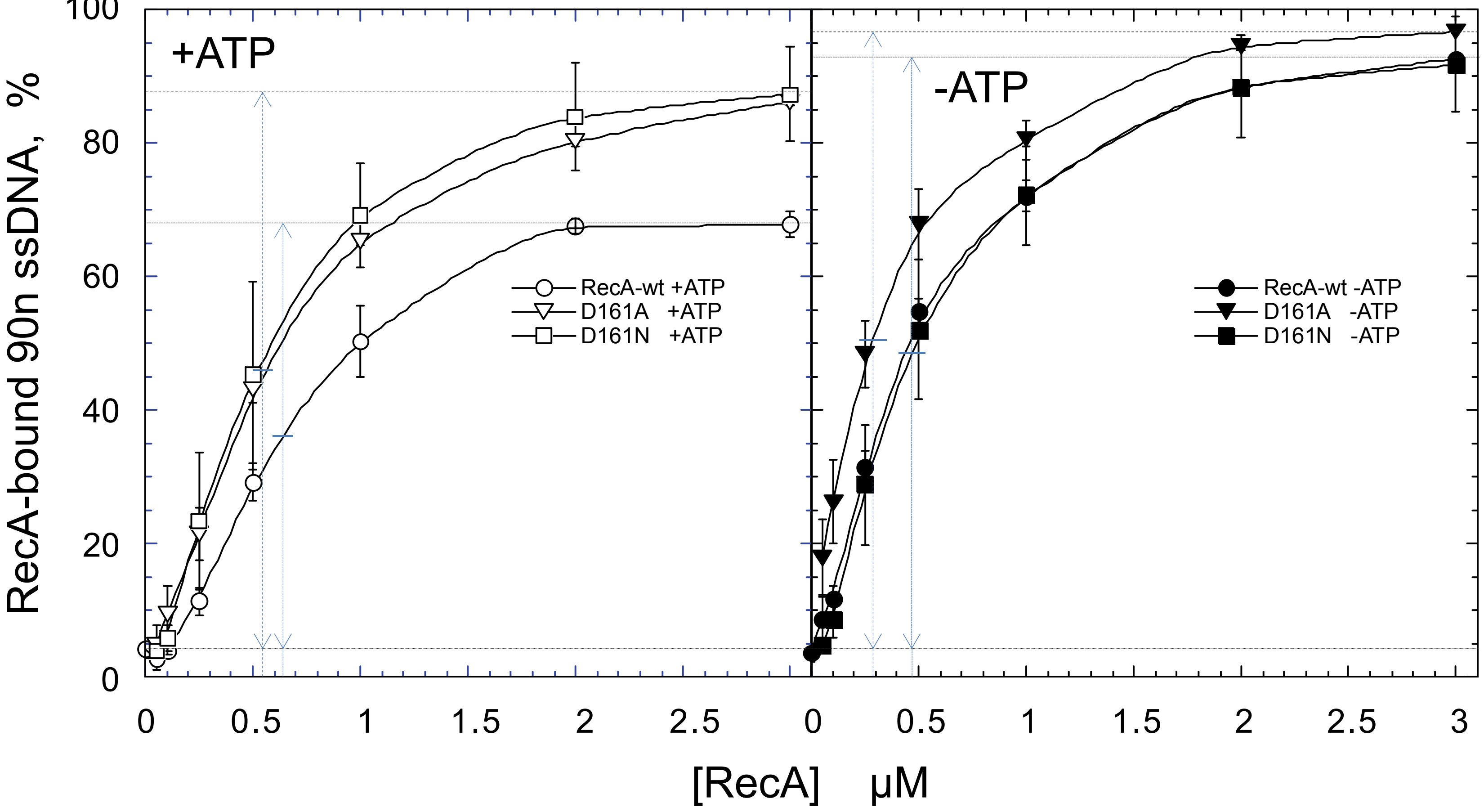


Figure S2

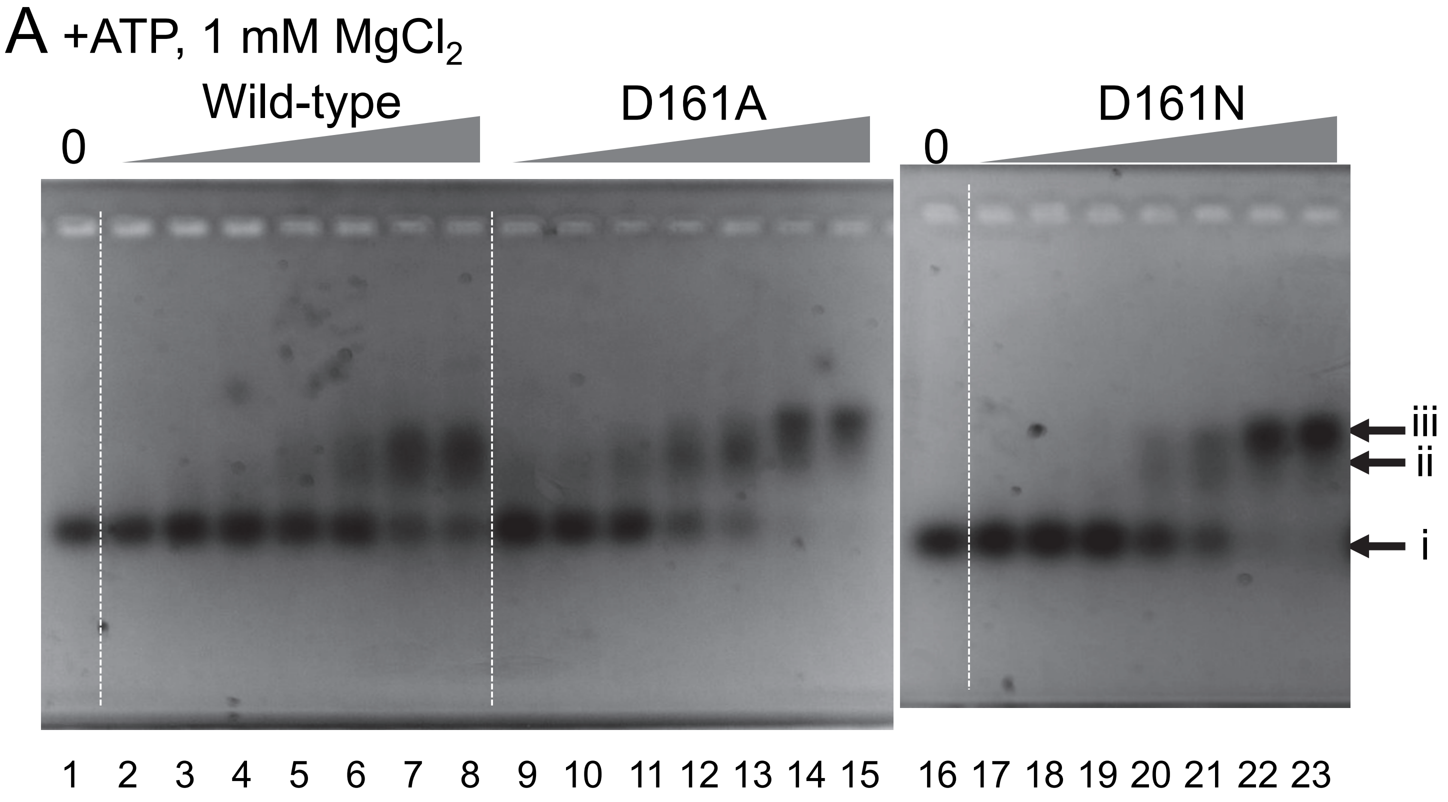
A +ATP, 1 mM MgCl2
Wild-type
D161A
D161N
0
0
iii
ii
i
1 2 3 4 5 6 7 8 9 10 11 12 13 14 15 16 17 18 19 20 21 22 23

Figure S3

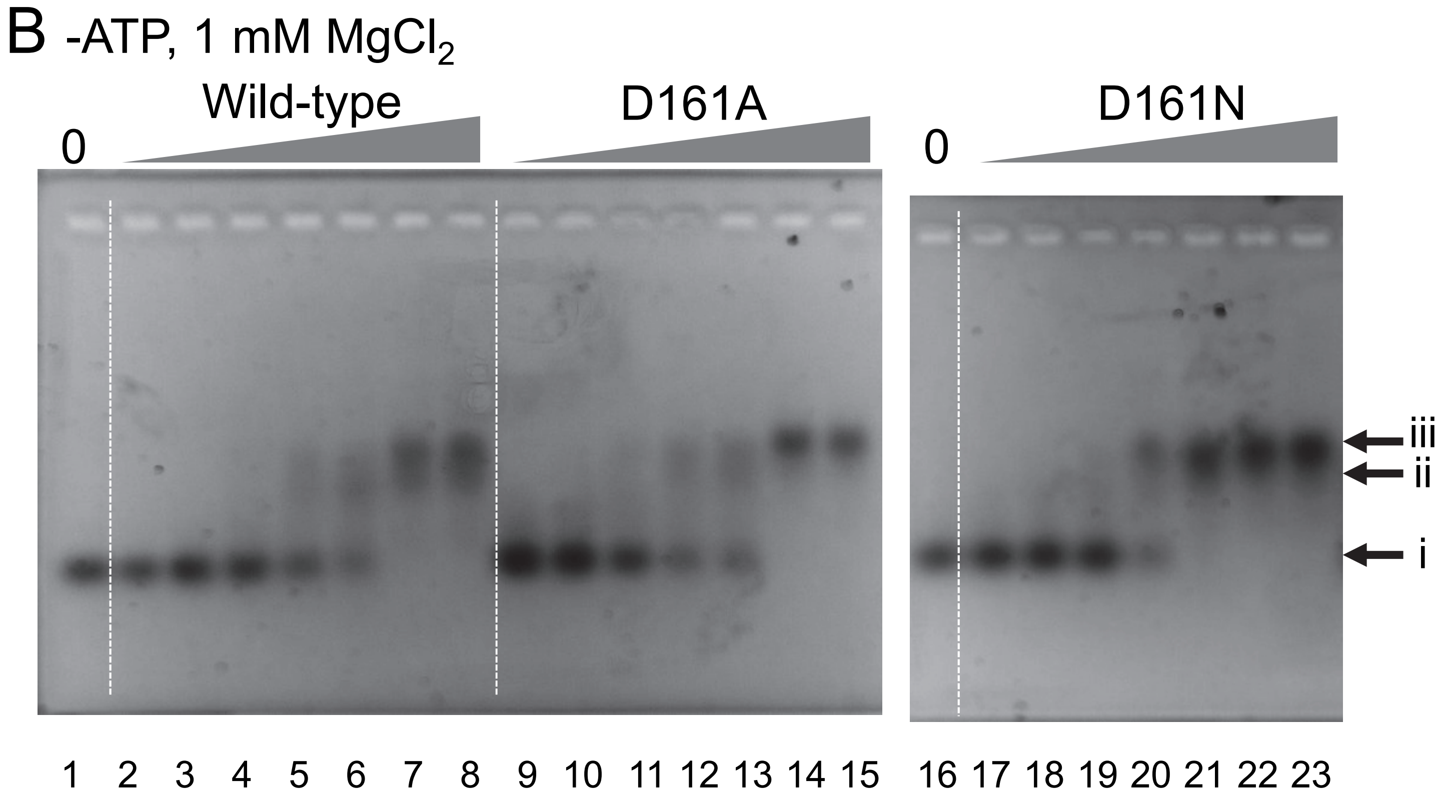
B -ATP, 1 mM $MgCl_2$
Wild-type
D161A
D161N
0
0
iii
ii
i
1 2 3 4 5 6 7 8 9 10 11 12 13 14 15 16 17 18 19 20 21 22 23

Figure S3

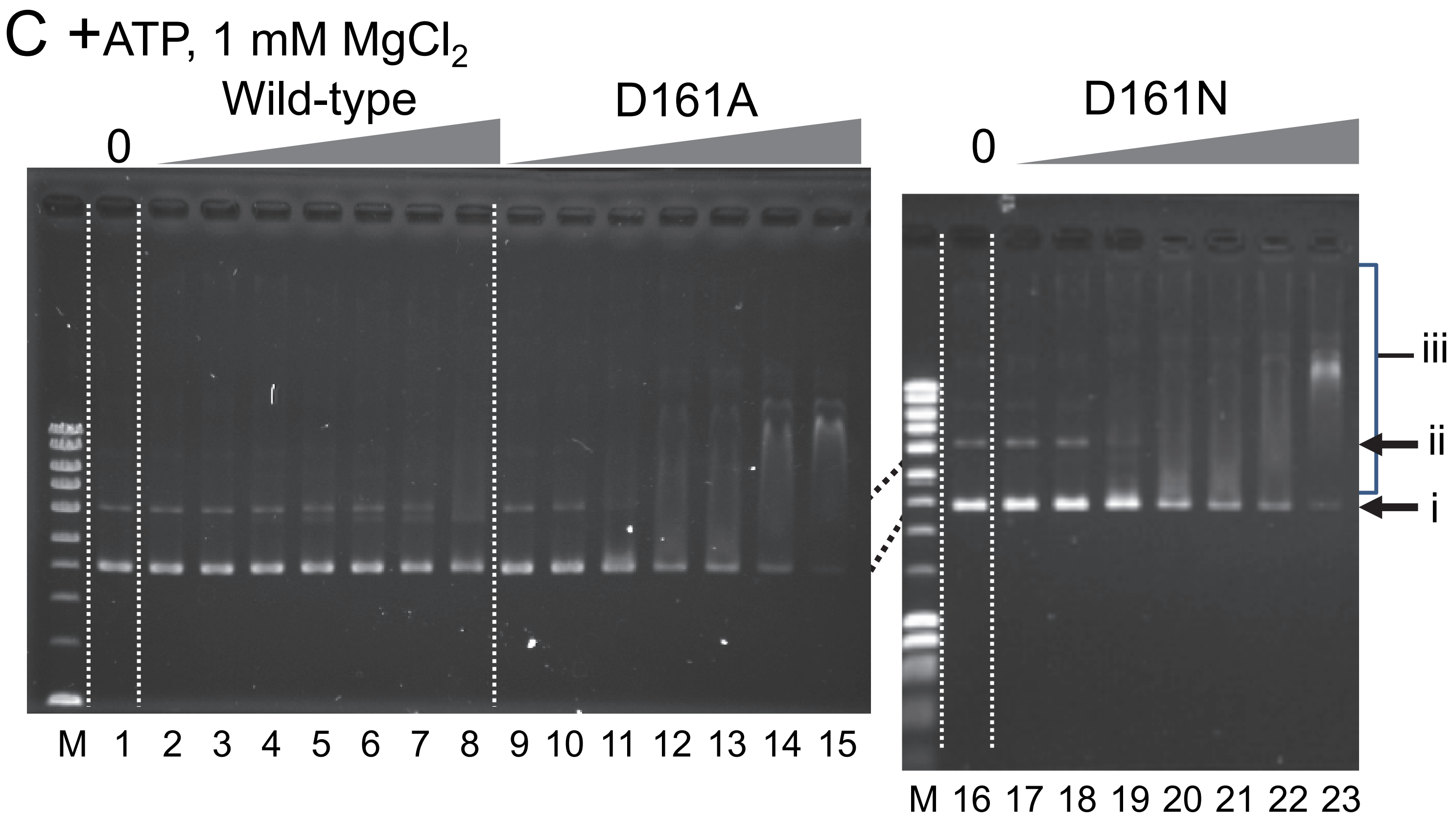
C +ATP, 1 mM MgCl2
Wild-type
D161A
D161N
0
0
iii
ii
i
M 1 2 3 4 5 6 7 8 9 10 11 12 13 14 15
M 16 17 18 19 20 21 22 23

Figure S3

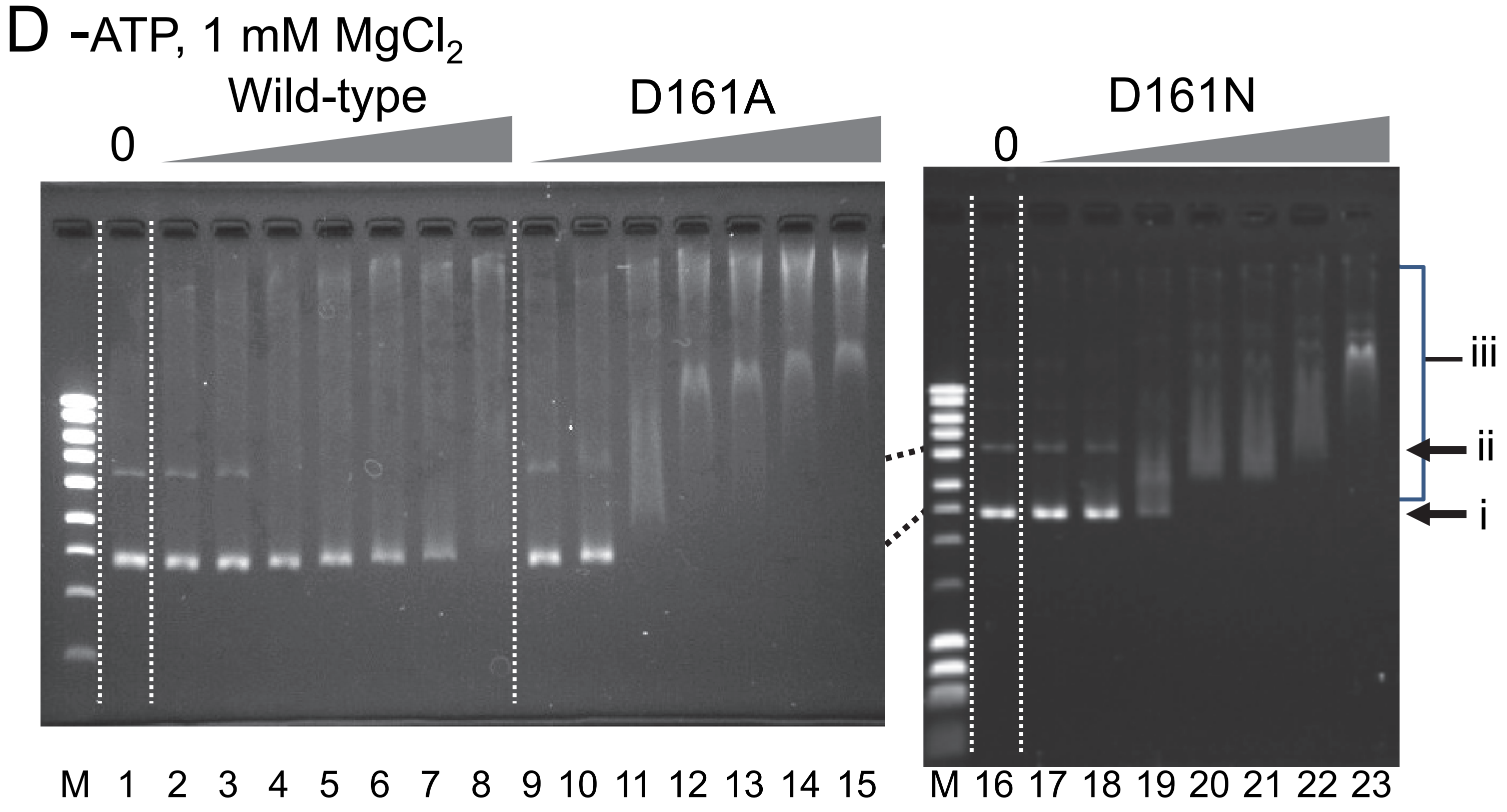
D -ATP, 1 mM MgCl2
Wild-type
D161A
D161N
0
0
iii
ii
i
M 1 2 3 4 5 6 7 8 9 10 11 12 13 14 15
M 16 17 18 19 20 21 22 23

Figure S3

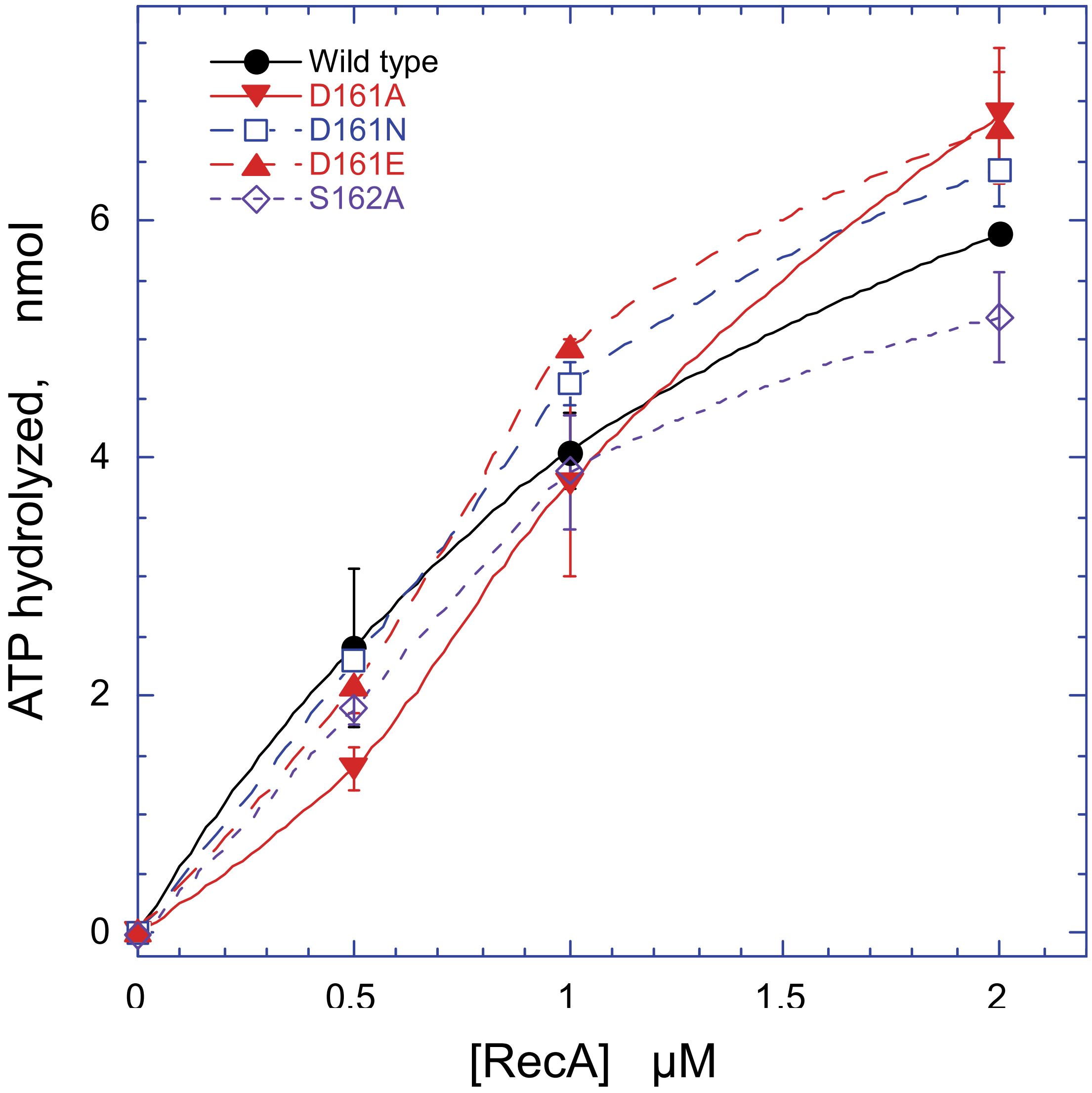

Wild type
D161A
D161N
D161E
S162A
ATP hydrolyzed, nmol
0
2
4
6
0
0.5
1
1.5
2
[RecA] μM


Figure S4

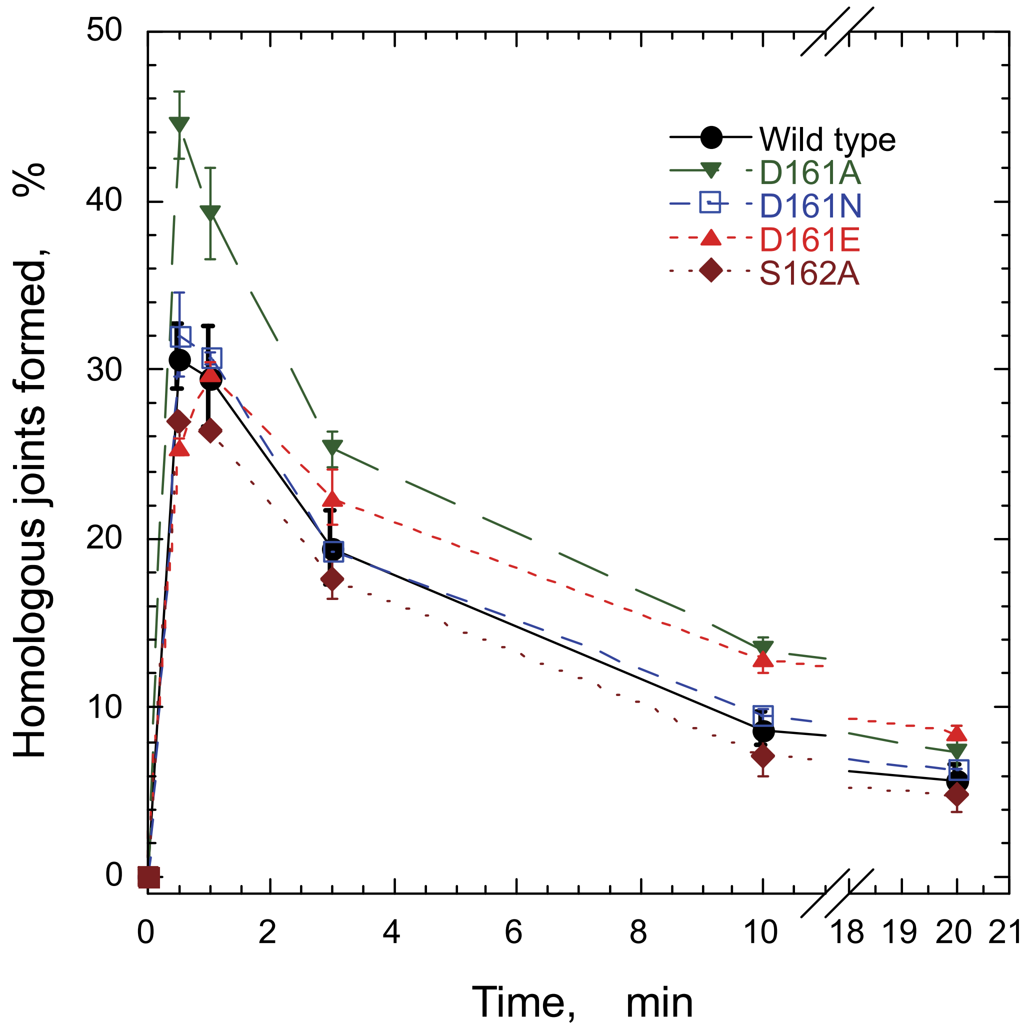

Wild type
D161A
D161N
D161E
S162A
Homologous joints formed, %
Time, min
0
10
20
30
40
50
0
2
4
6
8
10
18
19
20
21


Figure S5

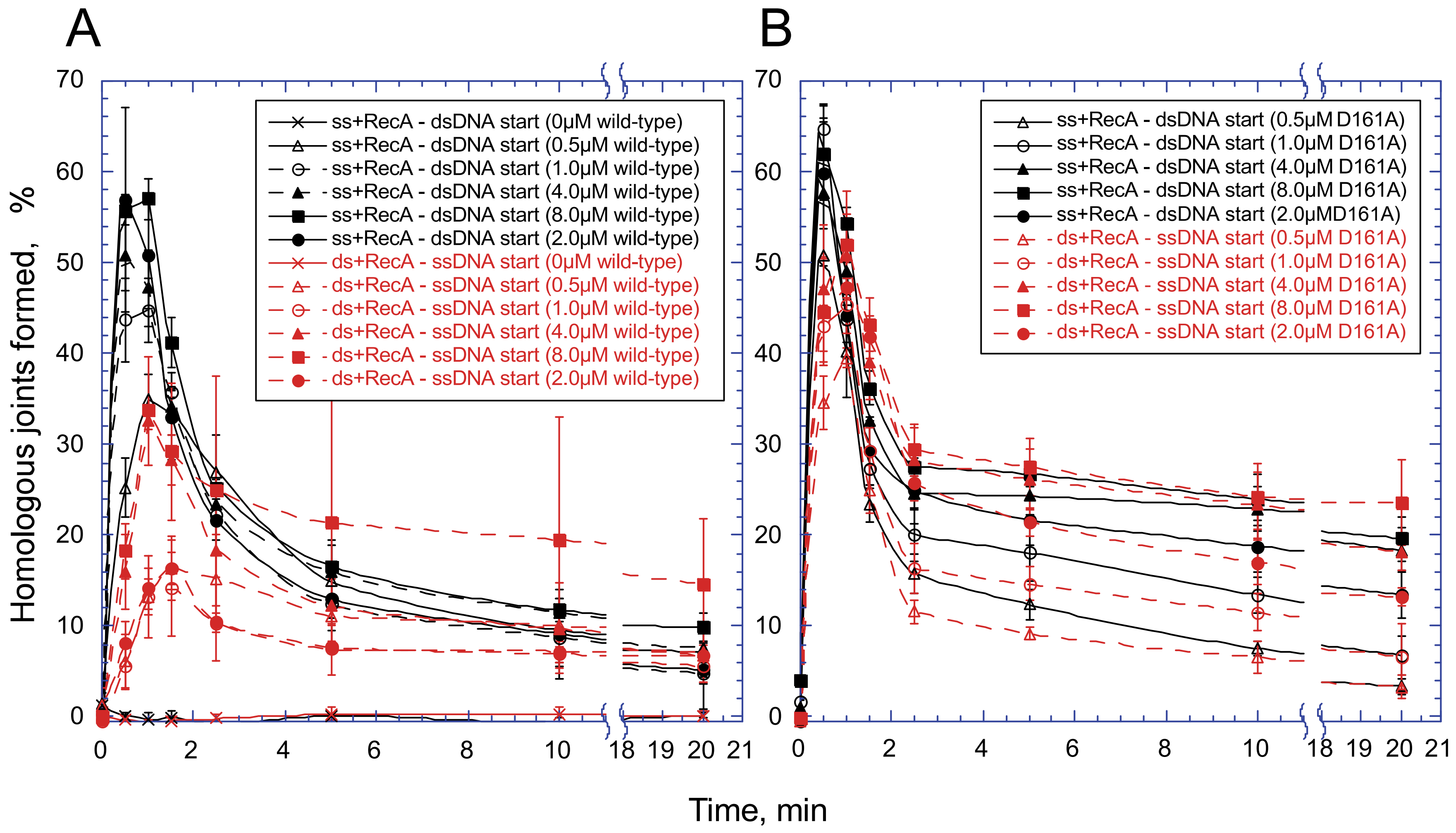
A
B
Homologous joints formed, %
Time, min
70
60
50
40
30
20
10
0
0
2
4
6
8
10
18
19
20
21
ss+RecA - dsDNA start (0µM wild-type)
ss+RecA - dsDNA start (0.5µM wild-type)
ss+RecA - dsDNA start (1.0µM wild-type)
ss+RecA - dsDNA start (4.0µM wild-type)
ss+RecA - dsDNA start (8.0µM wild-type)
ss+RecA - dsDNA start (2.0µM wild-type)
ds+RecA - ssDNA start (0µM wild-type)
ds+RecA - ssDNA start (0.5µM wild-type)
ds+RecA - ssDNA start (1.0µM wild-type)
ds+RecA - ssDNA start (4.0µM wild-type)
ds+RecA - ssDNA start (8.0µM wild-type)
ds+RecA - ssDNA start (2.0µM wild-type)
ss+RecA - dsDNA start (0.5µM D161A)
ss+RecA - dsDNA start (1.0µM D161A)
ss+RecA - dsDNA start (4.0µM D161A)
ss+RecA - dsDNA start (8.0µM D161A)
ss+RecA - dsDNA start (2.0µMD161A)
ds+RecA - ssDNA start (0.5µM D161A)
ds+RecA - ssDNA start (1.0µM D161A)
ds+RecA - ssDNA start (4.0µM D161A)
ds+RecA - ssDNA start (8.0µM D161A)
ds+RecA - ssDNA start (2.0µM D161A)

Figure S6

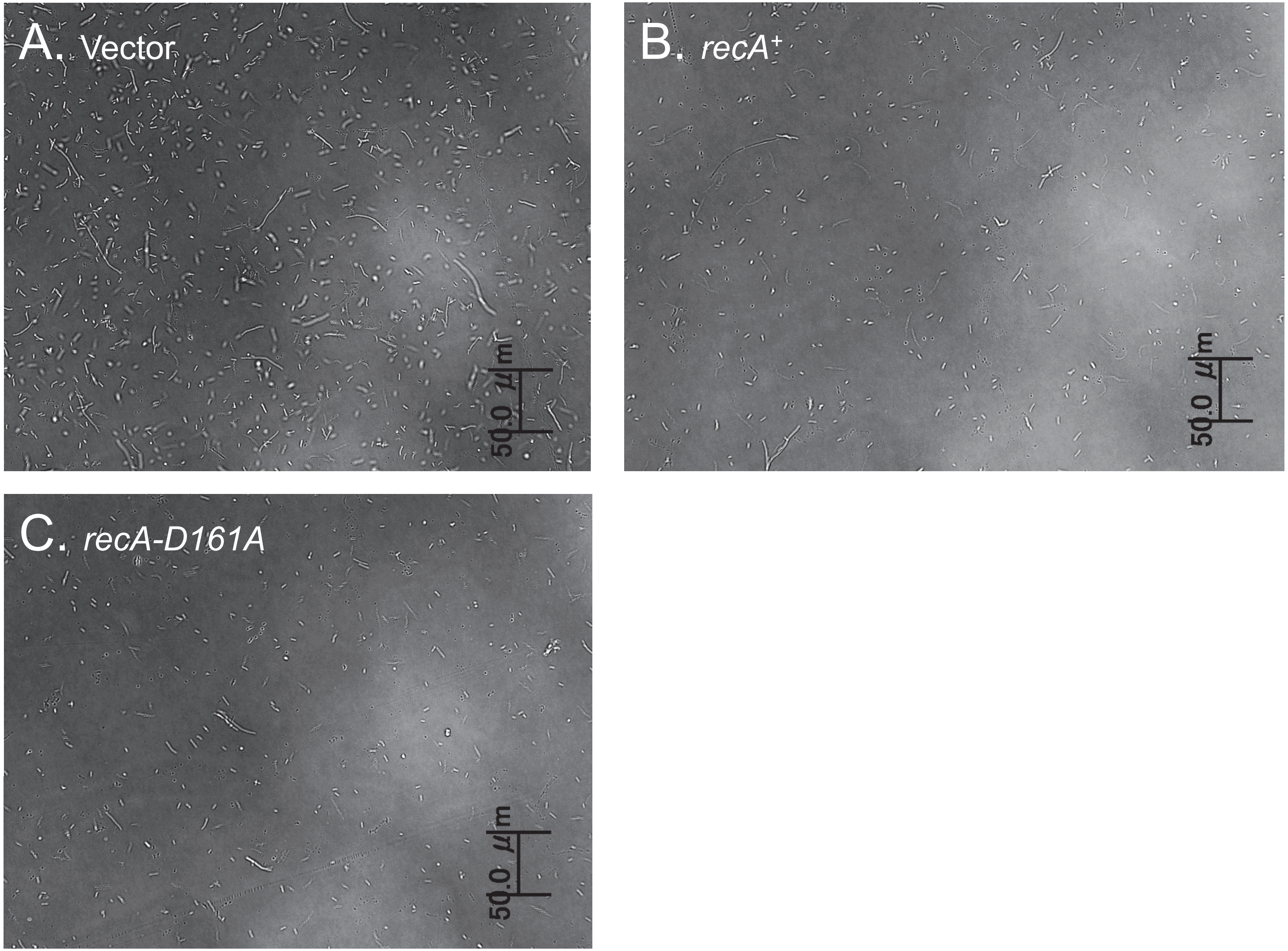


Figure S7

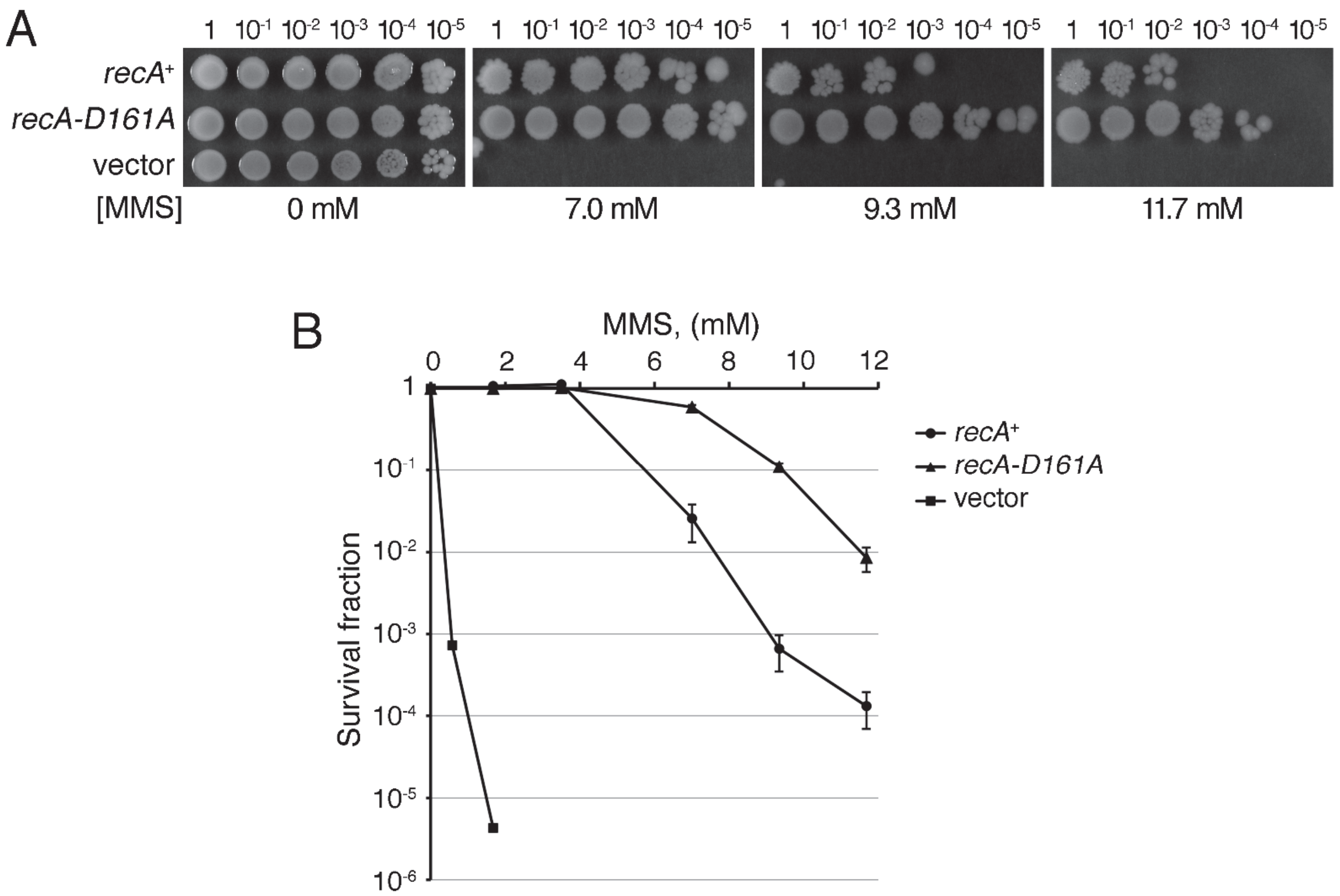
A
1 10-1 10-2 10-3 10-4 10-5
recA+
recA-D161A
vector
[MMS]
0 mM
7.0 mM
9.3 mM
11.7 mM
B
MMS, (mM)
0 2 4 6 8 10 12
Survival fraction
1
10-1
10-2
10-3
10-4
10-5
10-6
recA+
recA-D161A
vector

Figure S8

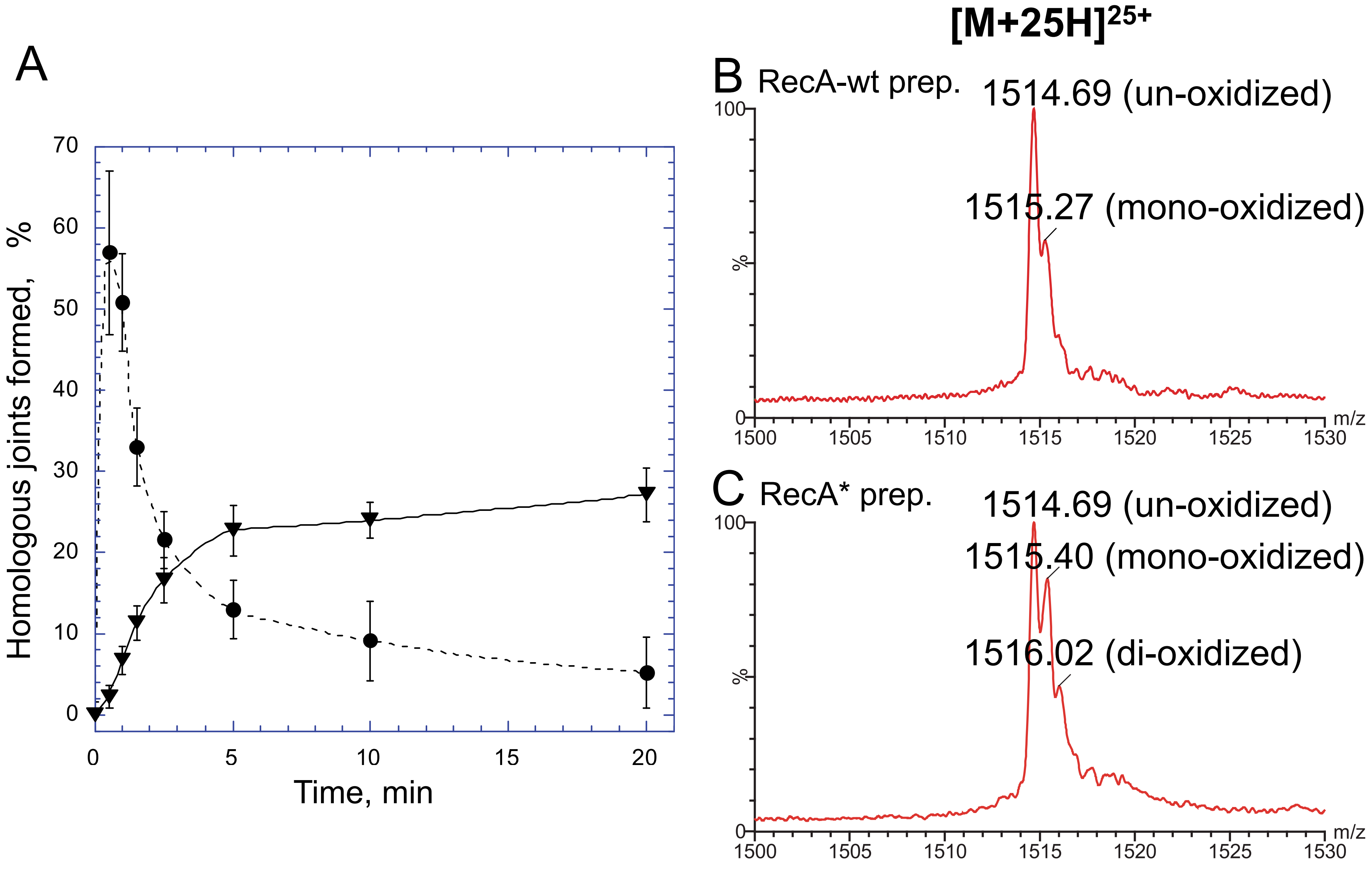

[M+25H]25+
A
Homologous joints formed, %
Time, min
0
5
10
15
20
0
10
20
30
40
50
60
70
B RecA-wt prep.
1514.69 (un-oxidized)
1515.27 (mono-oxidized)
C RecA* prep.
1514.69 (un-oxidized)
1515.40 (mono-oxidized)
1516.02 (di-oxidized)
%
m/z
100
0
1500
1505
1510
1515
1520
1525
1530


Figure S9
